# Common Fascial Abnormalities in Five Thumb Pain Conditions: A Fifteen-Point Ultrasound-Guided Fascia Hydrorelease Clinical Protocol

**DOI:** 10.3390/jfmk11030308

**Published:** 2026-08-04

**Authors:** Hiroaki Kimura, Ryoya Asaka, Tadashi Kobayashi, Hideaki Obata

**Affiliations:** 1 Kimura Pain Clinic, Maebashi 371-0013, Japan; pt.asaka63@gmail.com; 2 Development of Community Healthcare, Graduate School of Medicine, Hirosaki University, Hirosaki 036-8562, Japan; tkoba@hirosaki-u.ac.jp; 3 Department of Anesthesiology, Saitama Medical Center, Saitama Medical University, Kawagoe 350-8550, Japan; hoobata@gmail.com

**Keywords:** fascia hydrorelease, thumb pain, de Quervain disease, stacking fascia, ultrasound-guided treatment, fascial densification, hyaluronic acid, clinical protocol

## Abstract

**Background**: Thumb pain frequently arises from multiple conditions—de Quervain’s tenosynovitis, carpometacarpal (CMC) osteoarthritis, carpal tunnel syndrome, intersection syndrome, and myofascial pain syndrome—that often coexist yet are traditionally managed as separate entities. Fascial abnormalities, including hyaluronic acid-related densification and impaired interlayer gliding, have been proposed to constitute a shared pathological substrate underlying these conditions. Ultrasound-guided fascia hydrorelease (US-FHR) targeting fascial pathology has been used clinically for thumb–wrist pain; however, the anatomical targets and treatment regions have not been systematically described. **Aim**: This article presents a fifteen-point US-FHR clinical protocol for five converging thumb pain conditions, based on shared fascial abnormalities—densification and impaired interlayer gliding—that these conditions may have in common. It further provides a shared practical framework for pain physicians, orthopedic specialists, hand therapists, and acupuncturists. **Methods**: The protocol was organized based on a focused literature review on fascial biology, thumb–wrist anatomy, and the five converging conditions, combined with long-term clinical experience at Kimura Pain Clinic. For each POINT, the anatomical rationale, associated pain pattern, procedural concept, and major safety considerations were summarized. A four-direction screening test (thumb flexion, extension, abduction, and adduction) assessed in three modes (active contraction, resistance loading, and passive stretch), combined with nine established clinical tests, was integrated to identify the affected fascial structures. A diagnostic ultrasound finding characterized by multi-layered hyperechoic bands with reduced interlayer gliding—the sonographic appearance termed “stacking fascia” (a hyperechoic, stripe-shaped lesion), a previously described morphological sign that may reflect fascial densification—is described. **Results**: The protocol comprises 15 POINTs distributed across two approaches: nine POINTs via a dorsal approach and six POINTs via a palmar approach. The dorsal approach (9 POINTs) covers the first and third extensor compartments, their intersections (1st–2nd and 2nd–3rd), the two radial-artery crossing points (Points 5 and 6, where the radial artery passes deep to the first-compartment and EPL/ECRB–ECRL tendons, respectively), Gokoku (Hegu), the adductor pollicis, and the deep palmar arch. The palmar approach (6 POINTs) covers the thenar muscles, transverse carpal ligament, paraneural sheath and interfascicular epineurium of the median nerve, the median-nerve/FPL/FCR interface, the median nerve within the carpal tunnel, and the deeper palmar ligamentous structures adjacent to the median nerve. Periarterial release around the radial artery is proposed as a hypothesis-generating approach to CMC osteoarthritis, in which fascial constriction of periarterial tissue may contribute to subchondral vascular compromise. For each POINT, the anatomical rationale, associated pain patterns, procedural concept, and safety considerations are integrated and described. **Conclusions**: The proposed fifteen-point protocol represents an expanded, structured, and clinical-experience-based framework for US-FHR in five converging thumb pain conditions sharing common fascial abnormalities. It may serve as a practical basis for the standardization, education, and broader dissemination of US-FHR in this population. Prospective observational studies, randomized controlled trials, and imaging validation studies are needed to evaluate its clinical effectiveness and to test the underlying mechanistic hypotheses, including potential periarterial vascular contributions to CMC osteoarthritis.

## 1. Introduction

Thumb pain has been described as a significant clinical challenge due to its multifactorial etiology and the functional importance of the thumb in daily activities. The thumb is estimated to account for approximately 40% of hand function [1], and pain in this region can severely impair quality of life [2]. Traditionally, conditions affecting the thumb—including de Quervain’s tenosynovitis, carpometacarpal (CMC) osteoarthritis, carpal tunnel syndrome (CTS), intersection syndrome, and myofascial pain syndrome (MPS)—have been diagnosed and treated as separate entities [3,4].

However, clinical observation suggests that these conditions frequently coexist in the same patient, suggesting a common underlying fascial mechanism. This raises the possibility that thumb pain may be understood as a group of overlapping presentations sharing a fascial substrate, consistent with the framework previously discussed for closely related conditions in Japanese-language observations [5].

The fascia is a continuous network of connective tissue that envelops and connects muscles, tendons, nerves, and vessels throughout the body [6,7]. Recent advances in fascial science have demonstrated that pathological changes in the fascia—particularly densification characterized by aggregation of hyaluronic acid (HA) within the loose connective tissue layers—can cause pain, restricted mobility, and nerve entrapment [8,9]. Stecco et al. have shown that HA densification increases viscosity within fascial planes, reducing gliding capacity and generating nociceptive signals [10]. Densification is conceptually defined as a reversible, HA-mediated change in the loose connective tissue, distinct from irreversible collagen-based fibrosis [11]; however, hyperechoic findings on ultrasound alone cannot reliably distinguish between these two states, and dynamic ultrasound assessment of fascial gliding can serve as an adjunctive diagnostic tool.

Ultrasound-guided release of the myofascia (muscle fascia) was first described by Kimura et al. in 2014 [12,13]; in 2015, the technique was extended from the myofascia to fascia in general, and it was subsequently termed fascia hydrorelease (FHR), a designation adopted by the Japanese Non-Surgical Orthopedics Society (JNOS) in 2017 [12,13]. FHR is defined as “an injection technique for the release (separation and relaxation) of abnormal fasciae to improve the extensibility and sliding, in addition to having analgesic effects”, described as being “akin to peeling off thin stacking papers and separating fasciae themselves” [12,13]. This technique has gained increasing attention as a minimally invasive alternative to surgical intervention for various musculoskeletal conditions.

FHR has been applied to anatomical regions beyond the thumb. For example, our previous work reported improvements in range of motion and pain following FHR on the coracohumeral ligament in patients with frozen shoulder [14].

In this clinical protocol article, we present the fascial basis of five converging thumb pain conditions, describe a representative 15-point FHR treatment protocol targeting the shared fascial substrate (detailed in Section 3; see Videos S1–S15), and discuss the molecular mechanisms underlying fascial densification and its reversal by hydrorelease.

### 1.1. Fascial Involvement in Five Converging Thumb Pain Conditions

#### 1.1.1. Five Converging Pathologies and Their Common Fascial Abnormalities

The thumb region—encompassing the thumb through the radial aspect of the wrist—is affected by several common pain-generating conditions: de Quervain’s tenosynovitis, CMC osteoarthritis, carpal tunnel syndrome, intersection syndrome, and myofascial pain syndrome (MPS). Differential diagnoses for symptoms in this region include C6 radiculopathy, radial nerve entrapment at the spiral groove or supinator, and rarely, central causes such as cerebrovascular events. Despite comprehensive evaluation, cases frequently remain unexplained and prove refractory to treatment.

It should be noted that conditions causing symptoms in the thumb region extend beyond these five entities to include neuropathic pain (central nervous system lesions, C6 radiculopathy, and radial nerve palsy), osseous pathology (Colles fracture, scaphoid fracture, stress fractures, and Kienböck disease [lunate avascular necrosis]), traumatic ligament injury (ulnar collateral ligament [UCL] injury of the thumb), and systemic inflammatory diseases (rheumatoid arthritis and CPPD). In this clinical protocol article, we propose a pathological framework for the five anatomically based thumb conditions with fascia as their common key pathological substrate, fundamentally excluding the above conditions. However, these excluded conditions may coexist as comorbidities, or fascial abnormalities may persist following resolution of these primary diseases.

These conditions overlap considerably in their anatomical distribution, and from a fascial perspective, their pain mechanisms share much in common. Notably, ultrasound examination of these patients frequently reveals fascial abnormalities—particularly “stacking fascia”—a structural phenotype comprising compacted (densified) fascial layers, visualized as hyperechoic stripe-shaped lesions on ultrasound [12]—across multiple anatomical sites, irrespective of which primary diagnosis predominates [5]. This clinical observation suggests that these five conditions may share a common fascial substrate.

#### 1.1.2. Evidence for a Shared Fascial Substrate

**Figure 1:**
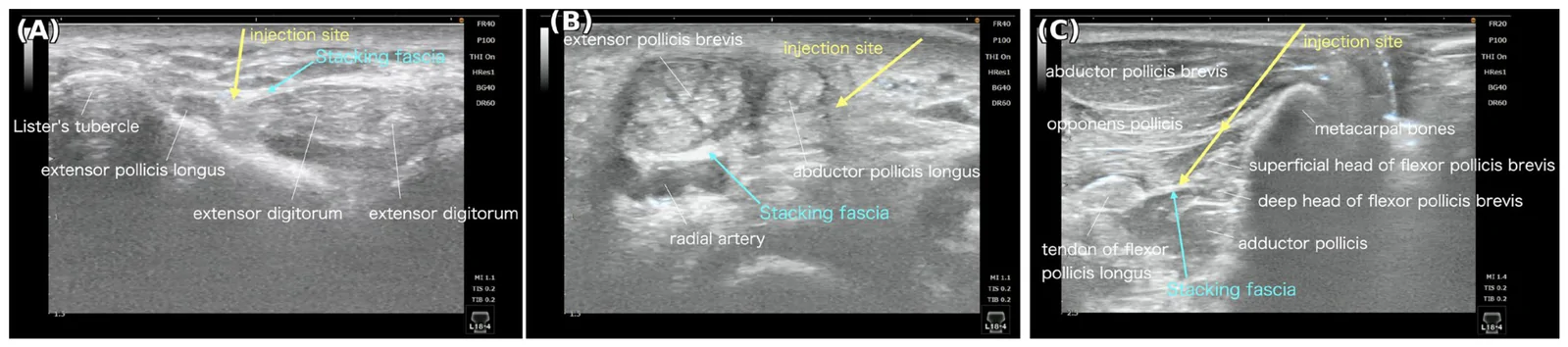
Representative ultrasound images of “stacking fascia” in the thumb–wrist region. Point numbers correspond to the 15-point treatment protocol described in Section 3. (**A**) Dorsal aspect in the third extensor compartment (Point 2): extensor pollicis longus (EPL) crossing the extensor digitorum tendons at the level of Lister’s tubercle. (**B**) Vascular crossing in the first extensor compartment (Point 5): radial artery passing adjacent to abductor pollicis longus (APL) and extensor pollicis brevis (EPB). (**C**) Palmar deep aspect in the thenar region (Point 10): layered thenar muscles (abductor pollicis brevis, opponens pollicis, superficial and deep heads of flexor pollicis brevis, and adductor pollicis) around the metacarpal bones, with the flexor pollicis longus (FPL) tendon visible. Cyan arrows indicate hyperechoic strip-shaped “stacking fascia”; yellow arrows indicate the injection site for ultrasound-guided fascia hydrorelease (US-FHR).

This shared imaging finding of stacking fascia—observed across all five conditions and at multiple anatomical sites in the thumb–wrist region (Figure 1)—suggests a possible unifying mechanism that may connect these pathologies.

This shared fascial substrate can be understood through the anatomical continuity of fascial planes in the thumb–wrist region, extending proximally into the forearm. The extensor retinaculum, which forms the six dorsal compartments, is continuous with the antebrachial fascia proximally and the deep fascia of the hand distally [15]. Similarly, the flexor retinaculum is continuous with the palmar fascia and the fascial sheaths of the thenar and hypothenar muscles [16]. Thus, fascial densification in one region can propagate along these continuous planes, affecting multiple structures simultaneously.

## 2. Materials and Methods

### 2.1. Diagnosis and Evaluation

#### 2.1.1. Clinical Presentation

Patients with these converging thumb pain conditions typically present with pain and functional impairment in the thumb region that does not conform neatly to a single diagnostic category. Physical examination may reveal the following:

- Positive Finkelstein test (suggesting de Quervain’s disease);
- Pain at the intersection of the first and second extensor compartments;
- Tenderness and crepitus at the CMC joint;
- Positive Phalen’s or Tinel’s test (suggesting CTS);
- Referred pain patterns from proximal trigger points.

**Figure 2:**
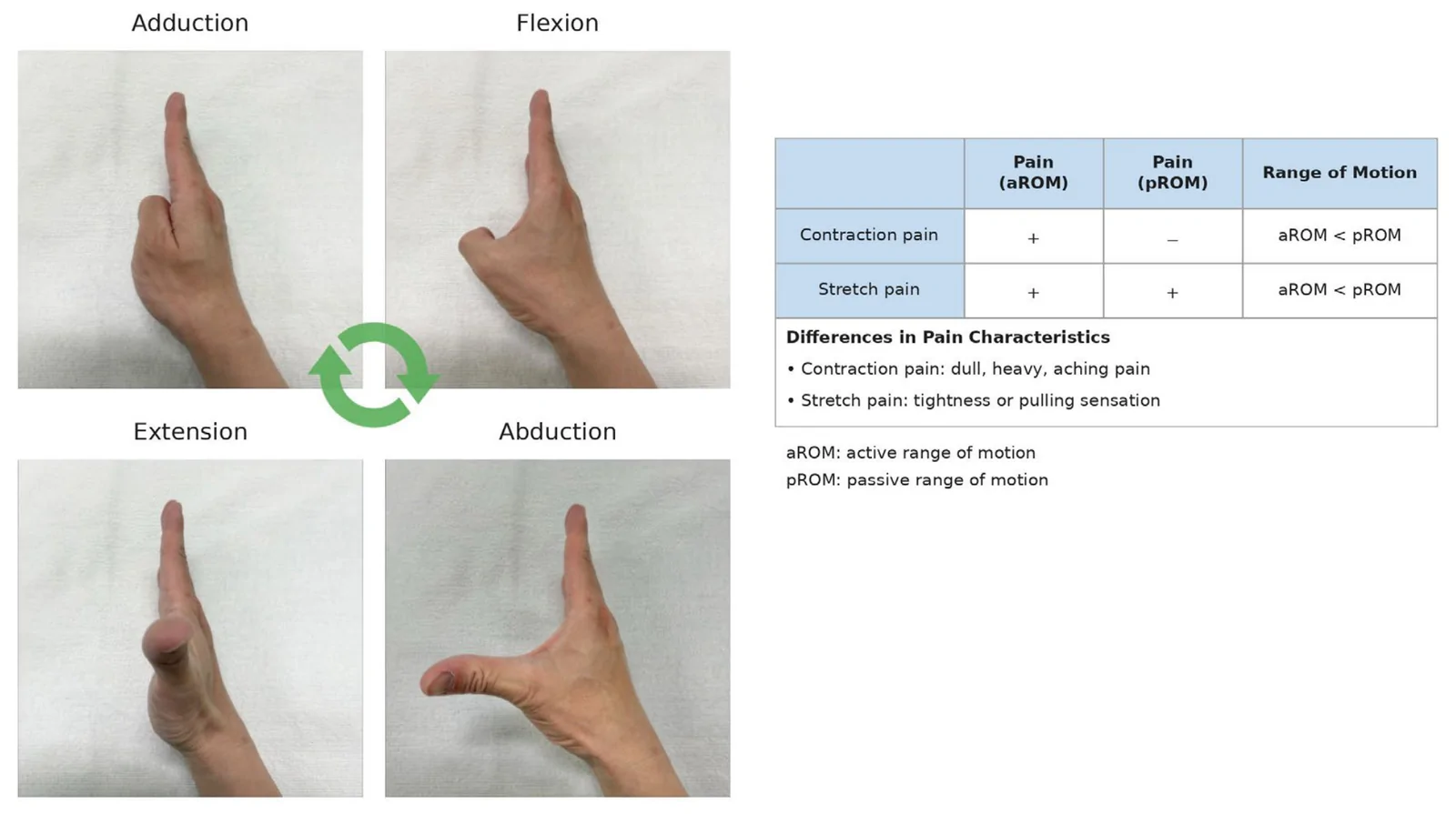
Screening test for five thumb pain conditions; four cardinal movement directions of the thumb (flexion, extension, abduction, and adduction) with the primary pain-generating fascial structures associated with each direction.

**Figure 3:**
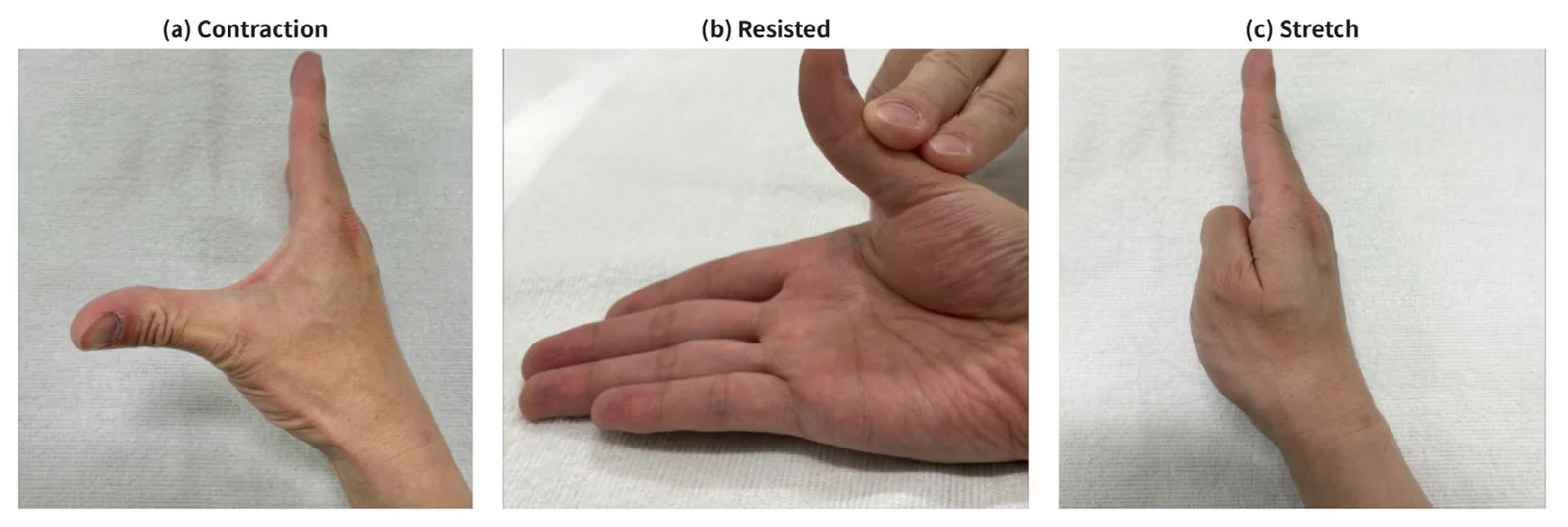
Detailed evaluation of five thumb pain conditions; using thumb abduction as an example, the three evaluation modes (active contraction, resistance loading, and passive stretch) are demonstrated. The same protocol is applied to all movement directions.

The screening test evaluates four cardinal movements of the thumb (Figure 2): flexion, extension, abduction, and adduction. Each movement is assessed in three modes (Figure 3)—active contraction, resistance loading, and passive stretch—to identify the affected fascial structures (Table 1). The diagnostic accuracy of common clinical tests and their interpretation from the fascial perspective are summarized in Table 2.

Importantly, patients with these converging conditions frequently demonstrate simultaneous positivity across multiple tests. This does not indicate the coexistence of multiple independent diseases but rather suggests that fascial densification is a common pathological substrate that elicits positive findings across multiple clinical tests [5,10]. This pattern of cross-test positivity may itself constitute a clinical observation consistent with—but not proof of—a shared fascial substrate; validation in prospective studies is required.

#### 2.1.2. Pain Sources Classified by Movement Direction

**Figure 4:**
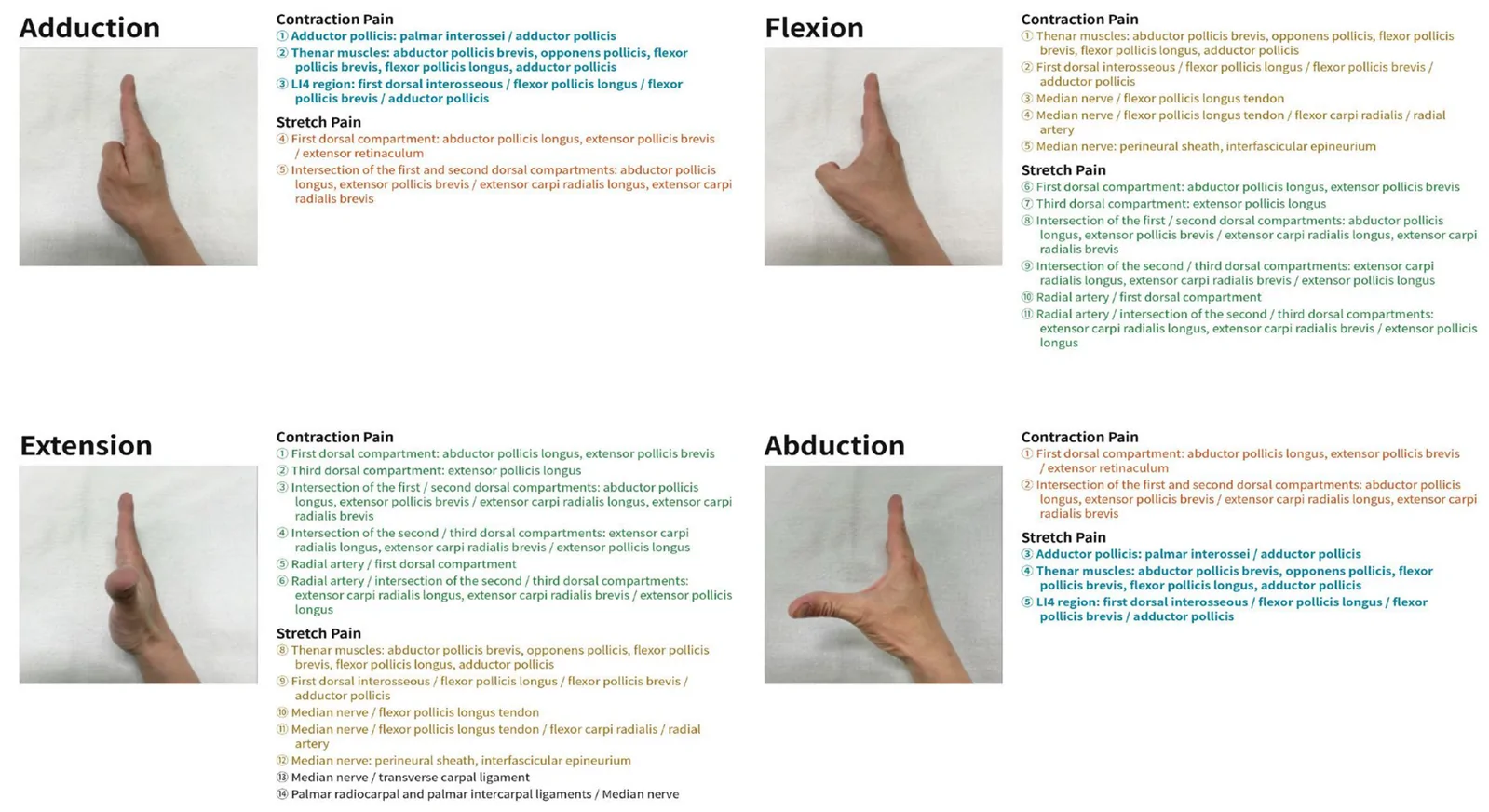
Movement-direction-specific pain source mapping: for each of the four cardinal directions (flexion, extension, abduction, and adduction), the anatomical structures requiring evaluation are listed in numbered order.

Understanding the relationship between thumb movement direction and specific pain sources is essential for targeted treatment (Figure 4). Based on the biomechanical principles of muscle contraction and stretching, pain sources in these five conditions can be systematically classified according to the direction of thumb movement that provokes pain. This classification distinguishes between contraction pain (pain arising from the actively contracting muscles and their fascial connections) and stretch pain (pain arising from structures being elongated by the movement). Table 3 presents this classification in detail.

We propose that contraction pain occurs when fascial densification in or around the agonist muscles impairs their normal contraction mechanics, building on prior observations that fascial densification disrupts gliding and generates nociceptive signals [10]. For example, during thumb adduction, contraction pain arises from the thenar muscles, first dorsal interosseous, and adductor pollicis, the primary adductors. During extension, the extensor compartments (first through third) become the pain sources.

This reciprocal pattern—herein proposed as a novel conceptual framework—also accounts for stretch pain: structures that are stretched during a given movement become pain sources when fascial adhesions restrict their normal elongation [10]. Thus, during thumb adduction, the first extensor compartment (antagonist to adduction) and the intersection of the first and second extensor compartments generate stretch pain. During extension, the flexor group—thenar muscles, median nerve complex, and flexor pollicis longus—becomes symptomatic as these structures resist elongation.

Several structures generate pain irrespective of movement direction: the extensor retinaculum, the deep palmar arch, and the metacarpal/trapezium articulation (CMC joint). These represent stress concentration sites [26] where mechanical forces converge from multiple directions, making them vulnerable to fascial densification independent of the specific movement pattern.

This movement-based classification directly informs the clinical application of the 15-point FHR protocol. By identifying which movements provoke pain and whether the pattern is consistent with contraction or stretch pain, the clinician can prioritize specific treatment points from the protocol, ensuring efficient and targeted fascial release.

### 2.2. Ultrasound-Guided Fascia Hydrorelease Technique

#### 2.2.1. Principles

Fascia hydrorelease (FHR) is an injection technique used to release abnormal fasciae (separation and relaxation) to improve extensibility and sliding, in addition to having analgesic effects [12,13]. The technique of ultrasound-guided FHR targets hyperechoic strip-shaped lesions on ultrasound images, akin to peeling off thin stacking papers and separating fasciae themselves [13]. FHR is performed under real-time ultrasound guidance using a high-frequency linear transducer (12–18 MHz). The basic principle involves the following steps:

1. Identification of the target fascial plane on ultrasound, recognizing “stacking fascia”—densified, multilayered fascial tissue—as hyperechoic stripe-shaped lesions with reduced gliding;
2. Needle insertion under ultrasound guidance (typically a 27G needle);
3. Injection of normal saline or extracellular fluid (e.g., bicarbonate Ringer’s solution) to mechanically separate the densified fascial layers [12,27];
4. Confirmation of fascial plane separation on ultrasound in real time.

In the proposed protocol, the volume of injectate is typically 1–2 mL per point, reflecting the small anatomical structures of the thumb–wrist region [12,13]. Injection depth is not standardized and varies among individuals depending on the target fascial layer and patient-specific anatomy; real-time ultrasound visualization allows precise depth confirmation in each case, with the depth scale displayed on the left side of each Supplementary Video (Videos S1–S15) providing direct visual reference. The duration over which fascial separation is maintained varies among individuals and depends on the chronicity and duration of the patient’s symptoms.

#### 2.2.2. Safety Considerations

The ultrasound-guided approach provides several safety advantages:

- Real-time visualization of the needle tip relative to critical structures (nerves and arteries);
- Ability to avoid direct injection into tendons, nerves, or vessels;
- Confirmation of correct needle placement before injection;
- Monitoring of injectate distribution.

For procedures involving peripheral nerves (e.g., median nerve hydrorelease), very fine needles (27G, 30G, or 32G) should be used, and attention must be paid to bevel orientation during insertion. Local anesthetics are not recommended for intraneural hydrorelease, as the therapeutic goal is fascial release rather than nerve blockade [13]. Key structures requiring particular attention include the radial artery (Points 5, 6, and 13), the median nerve (Points 11–15), and the deep palmar arch (Point 9). Standard aseptic technique should be observed. Caution is warranted in patients receiving anticoagulant or antiplatelet therapy and in the presence of local infection or skin lesions at the intended puncture site. Although no serious adverse events have been encountered in the authors’ practice, potential risks include vascular puncture with local hematoma, transient paresthesia, and inadvertent intraneural injection; the procedure should therefore be performed only by clinicians experienced in ultrasound-guided interventional techniques.

## 3. Results

The treatment approach for these converging thumb pain conditions depends on the underlying pathology. For intra-articular synovitis (inflammatory conditions), corticosteroid-containing intra-articular block injections may be performed. However, for the treatment of surrounding tissues, including the joint capsule, with fascia as the primary target, ultrasound-guided fascia hydrorelease (US-FHR) is indicated. Furthermore, as an intervention for aggravating factors, relearning of whole upper-limb movements related to the CMC joint and thumb motion—including forearm pronation/supination and upper arm to shoulder movements—is also important. In this way, by focusing on anatomical sites and pathology rather than being misled by disease names alone, accurate diagnosis and targeted treatment become possible. Consistent with the JNOS Fascial Pain Syndrome criteria, “stacking fascia” in this protocol is identified not by echogenicity alone but by the hyperechoic, stripe-shaped appearance with reduced interlayer gliding on dynamic scanning, together with reproduction of tenderness and restricted mobility at the same site [12].

The systematic treatment of these converging thumb pain conditions requires addressing fascial abnormalities at multiple anatomical sites. We have developed a representative 15-point protocol based on the fascial anatomy of the thumb–wrist region, organized from dorsal to palmar structures (Table 4). Notably, each of these 15 treatment points corresponds to one or more of the anatomical stress concentration site categories we have previously described [26] (seven of the eight categories in that framework apply to the thumb–wrist region): curved areas (joint capsules), cross-sections (muscle intersections), areas where multiple structures gather, tunnel structures (carpal tunnel), adipose tissue, perivascular and perineural fascia surrounding vessels and nerves (e.g., around the radial artery), and sites of accessory muscles. The thumb–wrist region is particularly rich in such stress concentration sites, which explains the convergence of multiple pathologies in this region. The FHR procedure for each point is demonstrated in the accompanying Supplementary Videos (Videos S1–S15). The correspondence between each point and its Supplementary Video is listed in Table 5.

The selection of these treatment points is grounded in the concept of “Eight Anatomical Stress Concentration Sites” that we proposed in a prior study [26]: (1) curved areas, (2) cross-sections, (3) areas where multiple structures gather, (4) areas around tunnel structures, (5) adipose tissue, (6) perivascular/perineural fascia surrounding vessels and nerves, (7) ligamentum flavum and epidural fascia, and (8) sites of accessory muscles. All 15 points in the thumb–wrist region correspond to one or more of these stress concentration categories, providing a theoretical basis for understanding why fascial densification preferentially occurs at these specific anatomical locations.

The point numbers represent a systematic anatomical classification rather than a prescribed treatment sequence. In clinical practice, the priority and selection of treatment points are determined by the patient’s pain location and clinical findings.

### 3.1. Selection Criteria for the Fifteen-Point Protocol

The fifteen POINTs described in this article were selected on the basis of four coordinated sources of information: (i) anatomical mapping of the thumb–wrist–forearm complex using standard anatomical references (including Berger 1997 [28] and Taleisnik 1976 [29] for the palmar radiocarpal and palmar intercarpal ligaments; Stecco et al. [6] for the fascial system); (ii) systematic sonographic observation of multi-layered hyperechoic bands with reduced interlayer gliding—the sonographic appearance termed “stacking fascia” (a hyperechoic, stripe-shaped lesion), a previously described morphological sign that may reflect fascial densification [12]—accumulated across patients presenting with each of the five converging conditions at Kimura Pain Clinic over more than a decade; (iii) clinical consensus derived from procedural experience across multiple operators and multiple presentations; and (iv) the published literature on the specific structures. No cadaveric dissection was performed by the authors specifically for the development of this protocol. It should be emphasized that most POINTs are supported by a combination of these sources rather than a single one, and that all POINTs, at the current stage, should be regarded as proposals derived from anatomical rationale, sonographic observation, clinical experience, and the published literature, rather than as definitive treatment targets validated by prospective controlled studies. The evidentiary basis for each of the fifteen POINTs is summarized in Table 6.

### 3.2. Dorsal Approach Releases

#### 3.2.1. Point 1: First Extensor Compartment Release (de Quervain)

The first extensor compartment contains the APL and EPB tendons, enclosed by the extensor retinaculum and a tendon sheath. Densification of the fascial layers surrounding these tendons is considered a central pathological feature in de Quervain’s disease. Under ultrasound guidance, saline is injected between the retinaculum and the tendon sheath to restore gliding.

**Anatomy:** APL (abductor pollicis longus), EPB (extensor pollicis brevis), retinaculum, and sheath.

**Indication:** de Quervain’s tenosynovitis and thumb pain on abduction/extension.

Pressure pain sites: (1) Directly over the extensor retinaculum, (2) the APL muscle belly, and (3) the EPB muscle belly.

Pain reproduction movements: (1) Thumb—radial abduction resistance; (2) thumb—MCP joint extension resistance from CMC joint slight abduction–flexion + IP joint slight flexion + MCP joint flexion position.

Orthopedic test: Finkelstein test positive.

**Figure 5:**
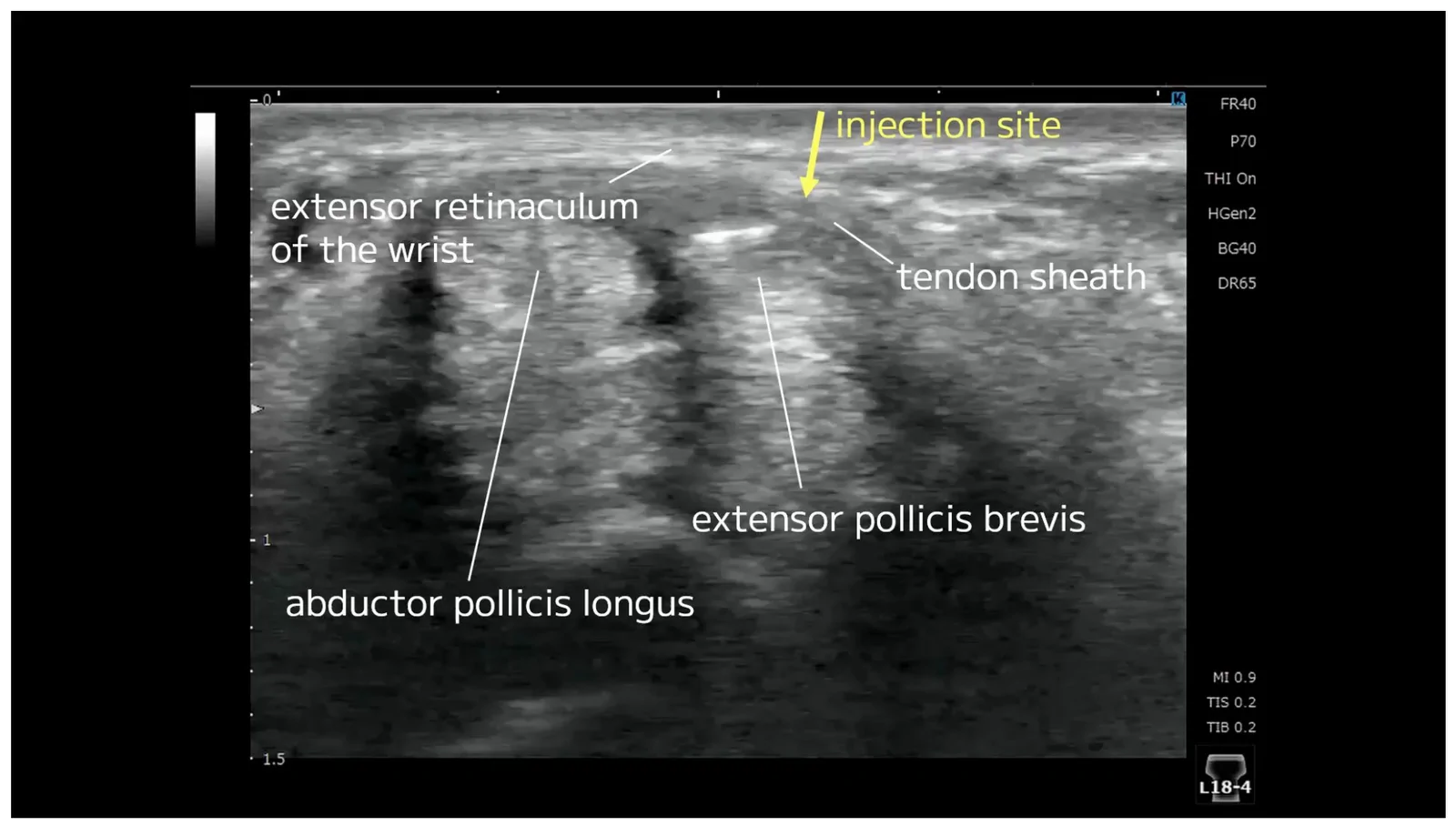
Point 1—first extensor compartment (APL/EPB): ultrasound-guided fascia hydrorelease; yellow arrow indicates the injection site.

Procedure (Video S1):

(1) Position the affected forearm in pronation with the dorsum facing up; the operator works from the affected side;
(2) Place the probe dorsally directly over the extensor retinaculum;
(3) Identify the stacking fascia on ultrasound and confirm the APL and EPB tendons;
(4) Insert the needle where tenderness is present and fascial stacking is confirmed on ultrasound;
(5) Perform US-FHR between the APL and EPB, releasing the stacking fascia (Figure 5).

#### 3.2.2. Point 2: Third Extensor Compartment Release (EPL)

The third extensor compartment contains the extensor pollicis longus (EPL) tendon, which passes around Lister’s tubercle of the radius. Fascial adhesion at this point can contribute to thumb extension weakness and pain.

**Anatomy:** EPL (extensor pollicis longus); Lister’s tubercle.

**Indication:** Pain on thumb extension, post-fracture stiffness.

Pressure pain sites: (1) EPL muscle belly; (2) Lister’s tubercle (over EPL tendon running on ulnar side).

Pain reproduction movements: Thumb—IP joint extension resistance.

**Figure 6:**
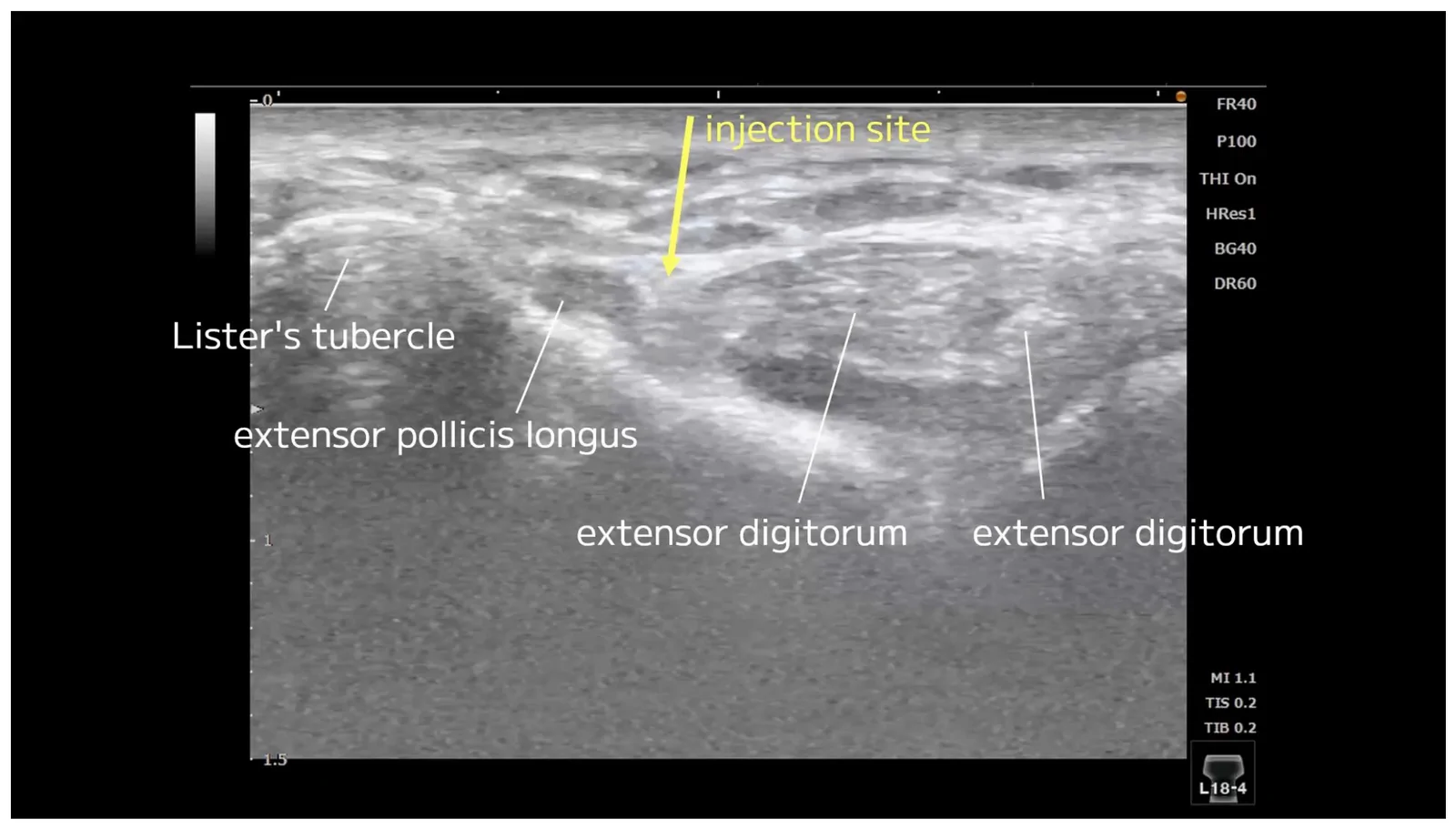
Point 2—third extensor compartment (EPL): ultrasound-guided fascia hydrorelease; yellow arrow indicates the injection site.

Procedure (Video S2):

(1) Position the forearm in pronation with the dorsum facing up;
(2) Place the probe dorsally directly over Lister’s tubercle;
(3) Identify the EPL tendon of the third extensor compartment lateral to Lister’s tubercle;
(4) Insert the needle where tenderness is present and fascial stacking is confirmed;
(5) Perform US-FHR to release the peri-EPL fascia (Figure 6).

#### 3.2.3. Point 3: Intersection of First and Second Compartments (APL-EPB/ECRL-ECRB)

**Figure 7:**
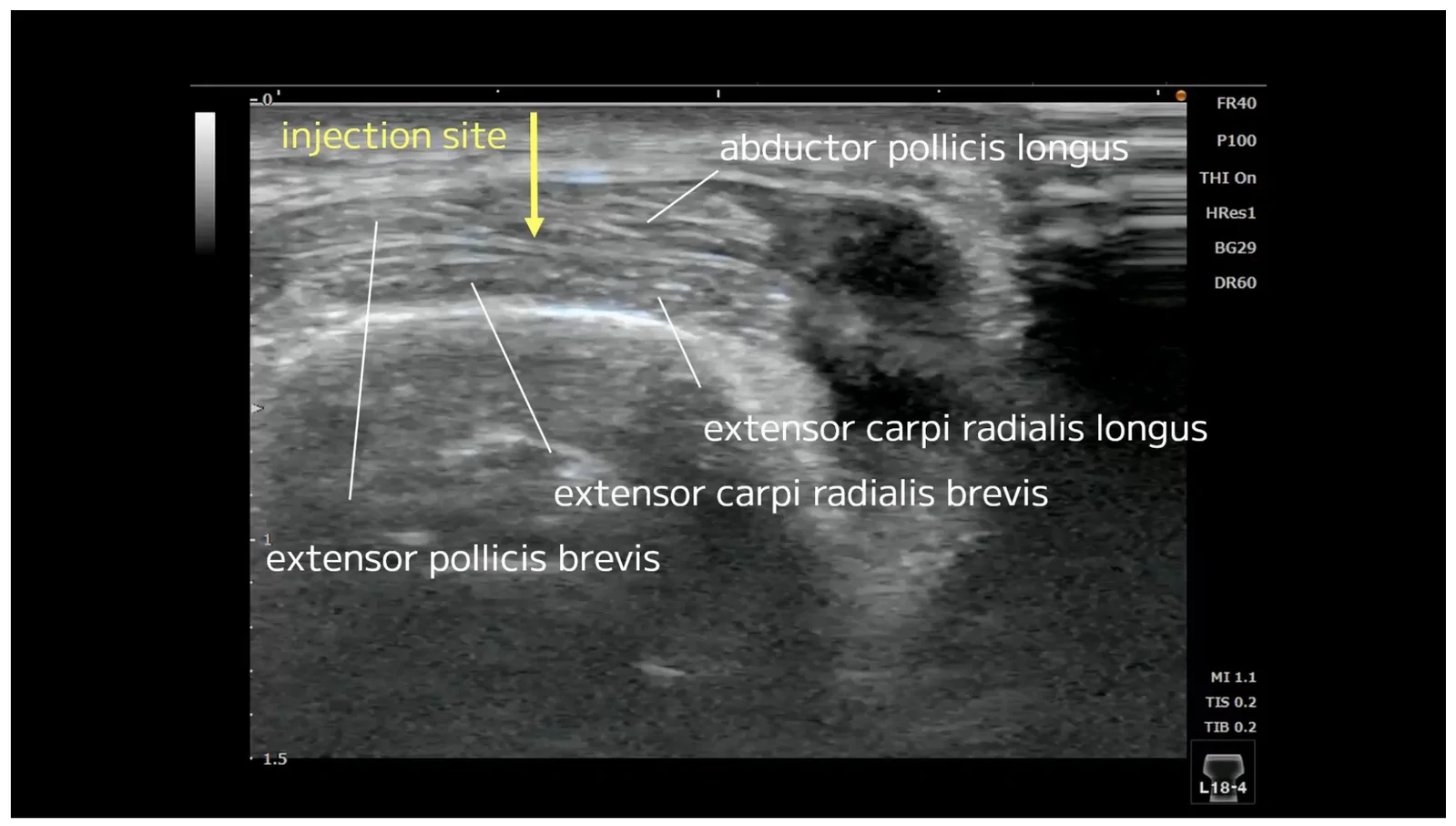
Point 3—first–second compartment intersection: ultrasound-guided fascia hydrorelease; yellow arrow indicates the injection site.

At the intersection where the first-compartment tendons (APL and EPB) cross over the second-compartment tendons (ECRL and ECRB), fascial friction and densification occur. This is the site of intersection syndrome. The dorsal approach at this intersection is illustrated in Figure 7.

**Anatomy:** APL and EPB crossing over the ECRL (extensor carpi radialis longus) and ECRB (extensor carpi radialis brevis), radius, and ulna.

**Indication:** Intersection syndrome, wrist motion pain, and thumb pain conditions.

Pressure pain sites: (1) APL/EPB muscle bellies (at crossing with ECRL/ECRB), (2) ECRL/ECRB muscle bellies, and (3) extensor retinaculum (directly over APL/EPB and ECRL/ECRB tendons).

Pain reproduction movements: (1) Thumb—radial abduction resistance and MCP joint extension resistance from CMC joint slight abduction–flexion + IP joint slight flexion + MCP joint flexion position; (2) wrist—radial deviation + palmar flexion resistance; and (3) wrist—dorsiflexion + radial deviation resistance.

Orthopedic test: Tenderness at the intersection site and crepitus during wrist motion.

Procedure (Video S3):

(1) Position the forearm in pronation with the dorsum facing up;
(2) Place the probe over the second dorsal compartment and identify the ECRL and ECRB tendons;
(3) Slide the probe ulnarly to identify the crossing point where the APL and EPB tendons from the first compartment cross over the second compartment;
(4) Insert the needle where fascial stacking is confirmed at the intersection of the first and second compartment tendons;
(5) Perform US-FHR between the APL/EPB and ECRL/ECRB.

#### 3.2.4. Point 4: Intersection of Second and Third Compartments (EPL/ECRB-ECRL)

A second intersection point exists where the EPL crosses the second compartment tendons. Fascial adhesion at this level contributes to combined thumb–wrist pain.

**Anatomy:** EPL, ECRB, and ECRL.

**Indication:** Thumb pain conditions, wrist motion pain, and lateral elbow pain.

Pressure pain sites: (1) ECRL/ECRB muscle bellies, (2) extensor retinaculum (over EPL tendon), (3) EPL muscle belly, and (4) Lister’s tubercle (over EPL tendon running on ulnar side).

Pain reproduction movements: (1) Wrist—dorsiflexion + radial deviation resistance (resistance applied to dorsal surface of index and middle metacarpals); (2) thumb—IP joint extension resistance.

**Figure 8:**
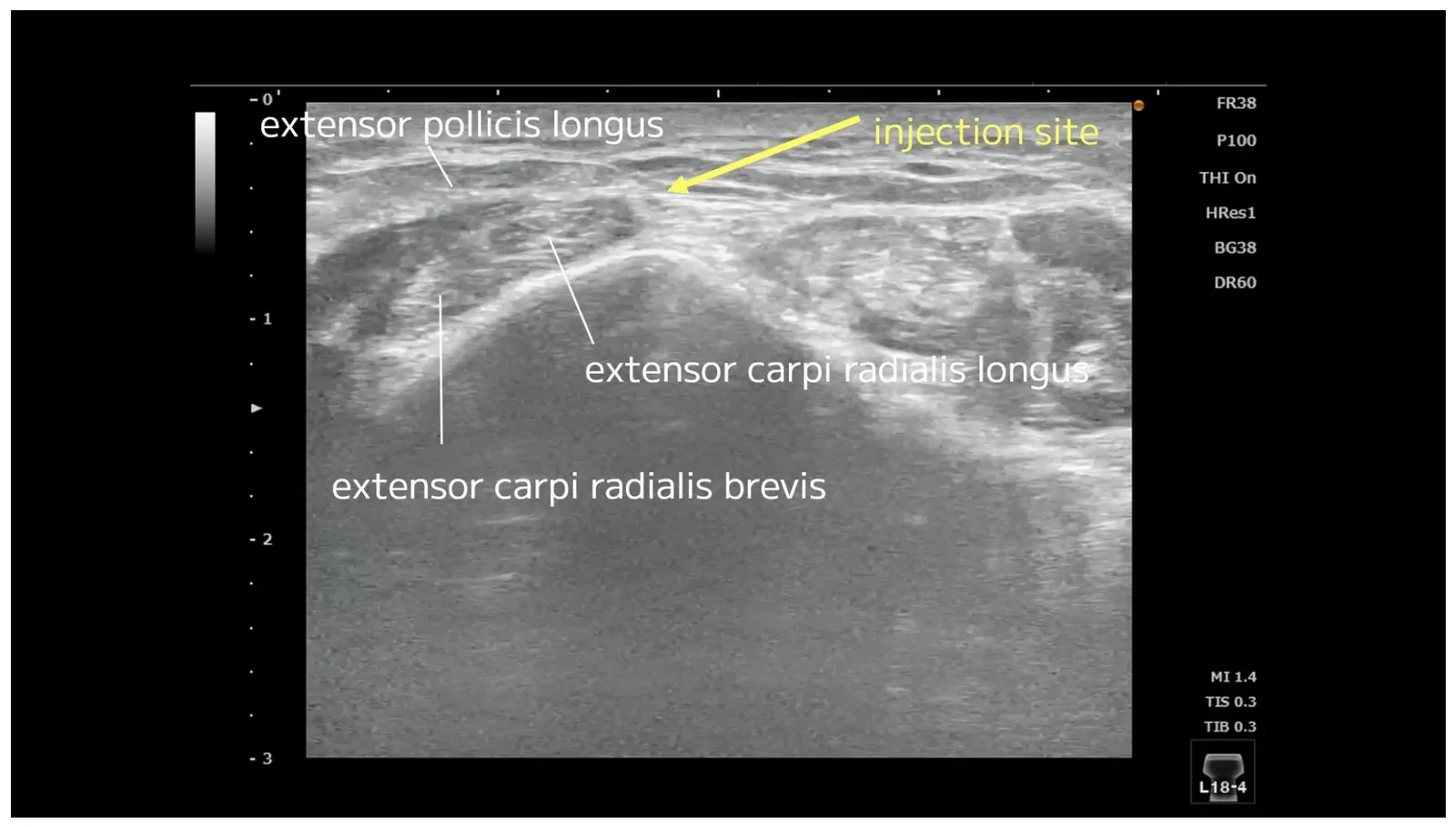
Point 4—second–third compartment intersection: ultrasound-guided fascia hydrorelease; yellow arrow indicates the injection site.

Procedure (Video S4):

(1) Position the forearm in pronation with the dorsum facing up;
(2) Place the probe over the second dorsal compartment to identify the ECRL and ECRB tendons;
(3) Slide the probe ulnarly to identify the crossing of the EPL from the third compartment;
(4) Insert the needle where fascial stacking is confirmed between the second and third compartment tendons;
(5) Perform US-FHR between the ECRL/ECRB and EPL (Figure 8).

#### 3.2.5. Point 5: Radial Artery/First Compartment Crossing (Vascular Release)

The radial artery crosses deep to the first extensor compartment (APL/EPB tendons) at the radial styloid level. Fascial adhesion at this vascular–tendinous crossing point creates a distinct pain generator that is separate from the de Quervain’s tendon sheath pathology. Ultrasound with color Doppler identifies the artery crossing the first compartment, and targeted hydrorelease at this interface may address the proposed vascular–fascial entrapment. This “vascular release” technique is proposed as an adjunctive approach for cases in which pain persists after conventional first-compartment treatment alone; controlled evidence remains lacking.

**Anatomy:** Radial artery, APL, EPB, first extensor compartment, and radial styloid.

**Indication:** Persistent radial wrist pain after de Quervain’s treatment, vascular–fascial entrapment in first compartment, and thumb pain on abduction/extension.

Pressure pain sites: (1) Crossing point of radial artery and first extensor compartment (APL/EPB); (2) periarterial tissue at radial styloid level.

Pain reproduction movements: (1) Thumb—radial abduction resistance; (2) thumb—MCP joint extension resistance from CMC joint slight abduction–flexion + IP joint slight flexion + MCP joint flexion position.

Ultrasound assessment: Color Doppler to confirm radial artery course and relationship with first-compartment tendons.

**Figure 9:**
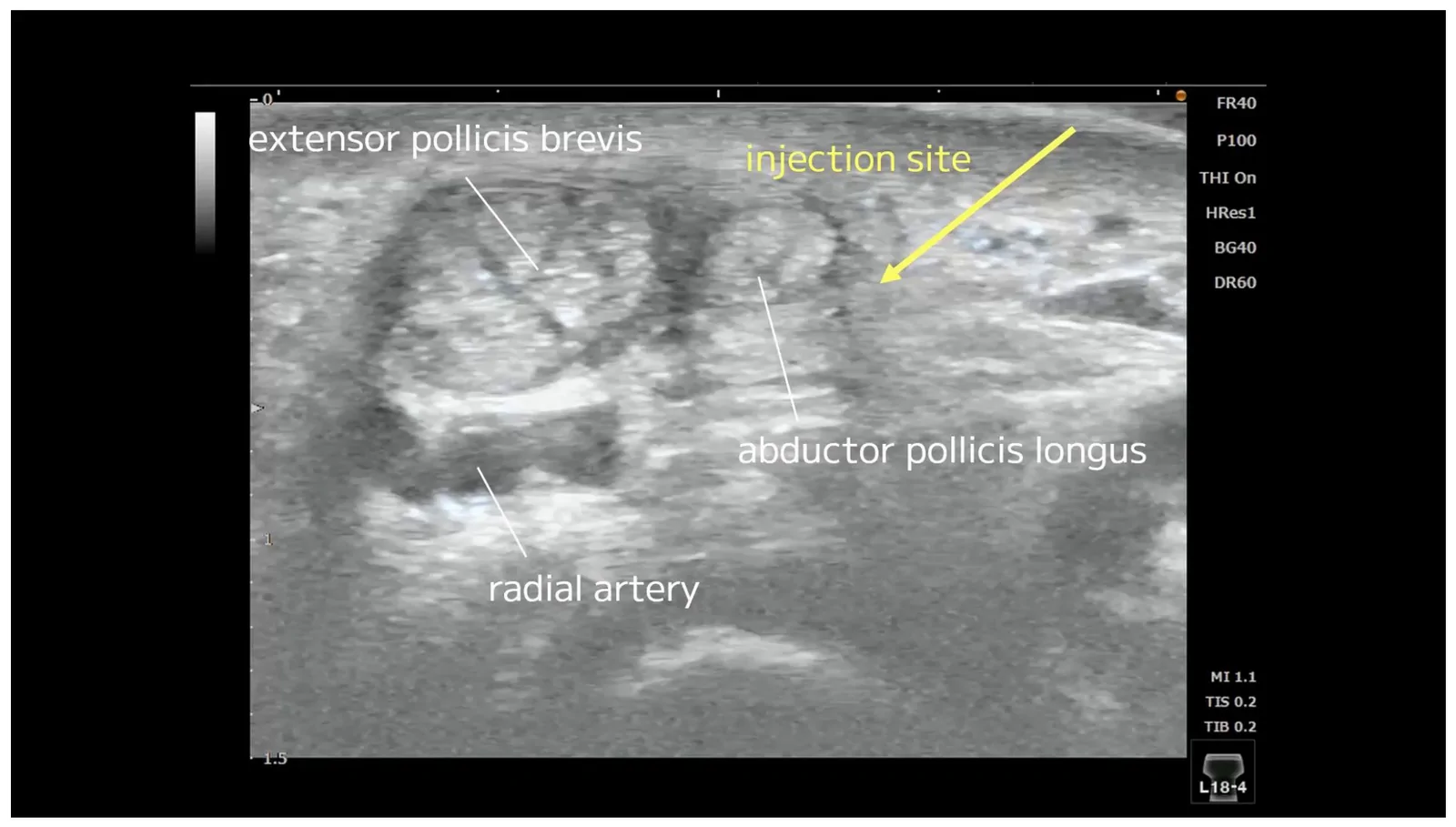
Point 5—radial artery/first compartment crossing: ultrasound-guided fascia hydrorelease; yellow arrow indicates the injection site.

Procedure (Video S5):

(1) Position the forearm in pronation with the dorsum facing up;
(2) Place the probe over the first extensor compartment at the level of the radial styloid;
(3) Use color Doppler to confirm the course of the radial artery and its relationship with the first-compartment tendons (APL/EPB);
(4) Insert the needle where tenderness is present at the artery–tendon crossing and fascial stacking is confirmed;
(5) Carefully perform US-FHR of the periarterial fascia, taking care to avoid arterial injury (Figure 9).

#### 3.2.6. Point 6: Radial Artery/EPL-ECRB-ECRL Crossing (Vascular Release)

The radial artery crosses deep to the extensor pollicis longus (EPL) tendon at the level of the anatomical snuffbox. Additionally, at this level, the EPL intersects with the second-compartment tendons (ECRL and ECRB), and the radial artery crosses deep to the EPL, ECRL, and ECRB. This vascular–tendon crossing is anatomically distinct from Point 5 (radial artery/first-compartment crossing) and may constitute an independent pain source. Fascial adhesion between the EPL tendon and the radial artery may contribute to pain during thumb extension and vascular–fascial entrapment. Using color Doppler to confirm the course of the radial artery in the snuffbox, fascial release is performed at the EPL crossing.

**Anatomy:** Radial artery (snuffbox portion), EPL tendon, anatomical snuffbox, and ECRL and ECRB tendons.

**Indication:** Snuffbox pain, radial wrist pain during thumb extension, and residual symptoms around the radial artery after Point 5 release.

Pressure pain sites: (1) Crossing point of EPL tendon and radial artery in the anatomical snuffbox, (2) radial aspect of EPL tendon, and (3) crossing of the third-compartment tendon (EPL) and second-compartment tendons (ECRL and ECRB).

Pain-reproducing movements: (1) Thumb—resisted IP joint extension; (2) wrist—radial deviation + dorsiflexion resistance.

Orthopedic examination: Snuffbox tenderness (differential diagnosis with scaphoid fracture required).

Ultrasound assessment: Color Doppler to confirm radial artery course in the snuffbox and its relationship with EPL tendon and evaluate arterial pulsation and surrounding fascial stacking.

**Figure 10:**
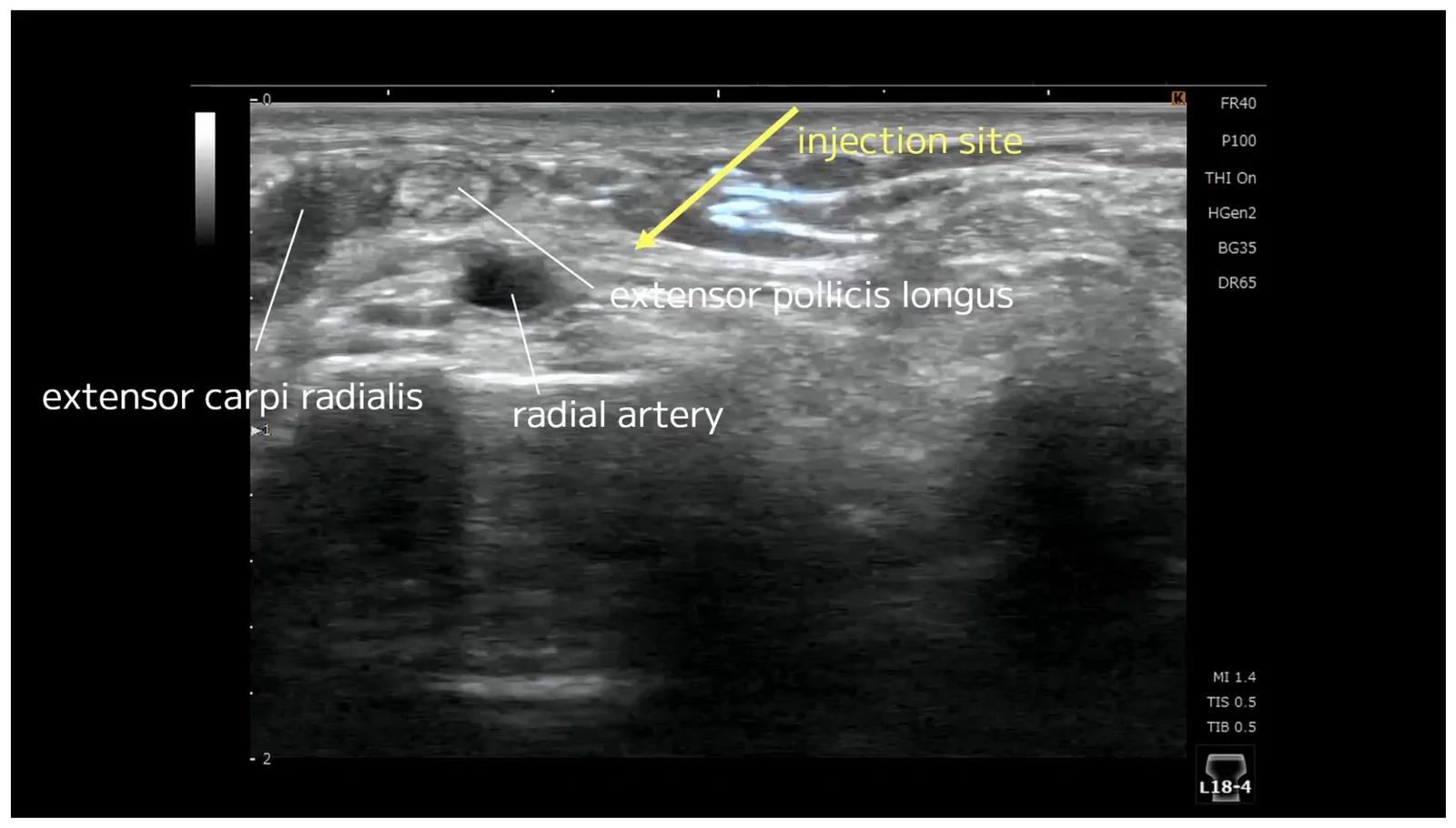
Point 6—radial artery/EPL–ECRL–ECRB crossing (snuffbox): ultrasound-guided fascia hydrorelease; yellow arrow indicates the injection site.

Procedure (Video S6):

(1) With the forearm in pronation (dorsum of the affected hand facing up), the operator works from the affected side;
(2) Place the probe over the anatomical snuffbox;
(3) Use color Doppler to confirm the course of the radial artery in the snuffbox and its relationship with the EPL tendon and ECRL and ECRB tendons;
(4) Insert the needle where tenderness is present at the radial artery–EPL/ECRL/ECRB crossing and fascial stacking is confirmed on ultrasound;
(5) Carefully perform US-FHR of the fascia between the radial artery and EPL/ECRL/ECRB tendons, taking care to avoid arterial injury (Figure 10).

#### 3.2.7. Point 7: Gokoku (Hegu)—First Dorsal Interosseous/Adductor Pollicis/Deep Palmar Arch and FPL

In the dorsal aspect of the first web space (corresponding to the acupuncture point “Hegu”/LI-4), the first dorsal interosseous muscle, adductor pollicis, and FPL tendon converge around the second metacarpal. Additionally, the deep palmar arch runs along the palmar aspect of the adductor pollicis. Fascial densification in this region causes deep thumb pain and web space contracture.

**Anatomy:** First dorsal interosseous, second metacarpal, adductor pollicis, FPL tendon, and deep palmar arch.

**Indication:** Deep thumb pain and web space contracture.

Pressure pain sites: Acupuncture point “Hegu” (LI-4)—dorsal web space between the thumb and index finger.

Pain reproduction movements: (1) Index finger—abduction resistance; (2) thumb—ulnar adduction resistance, palmar adduction resistance, and IP joint flexion resistance.

**Figure 11:**
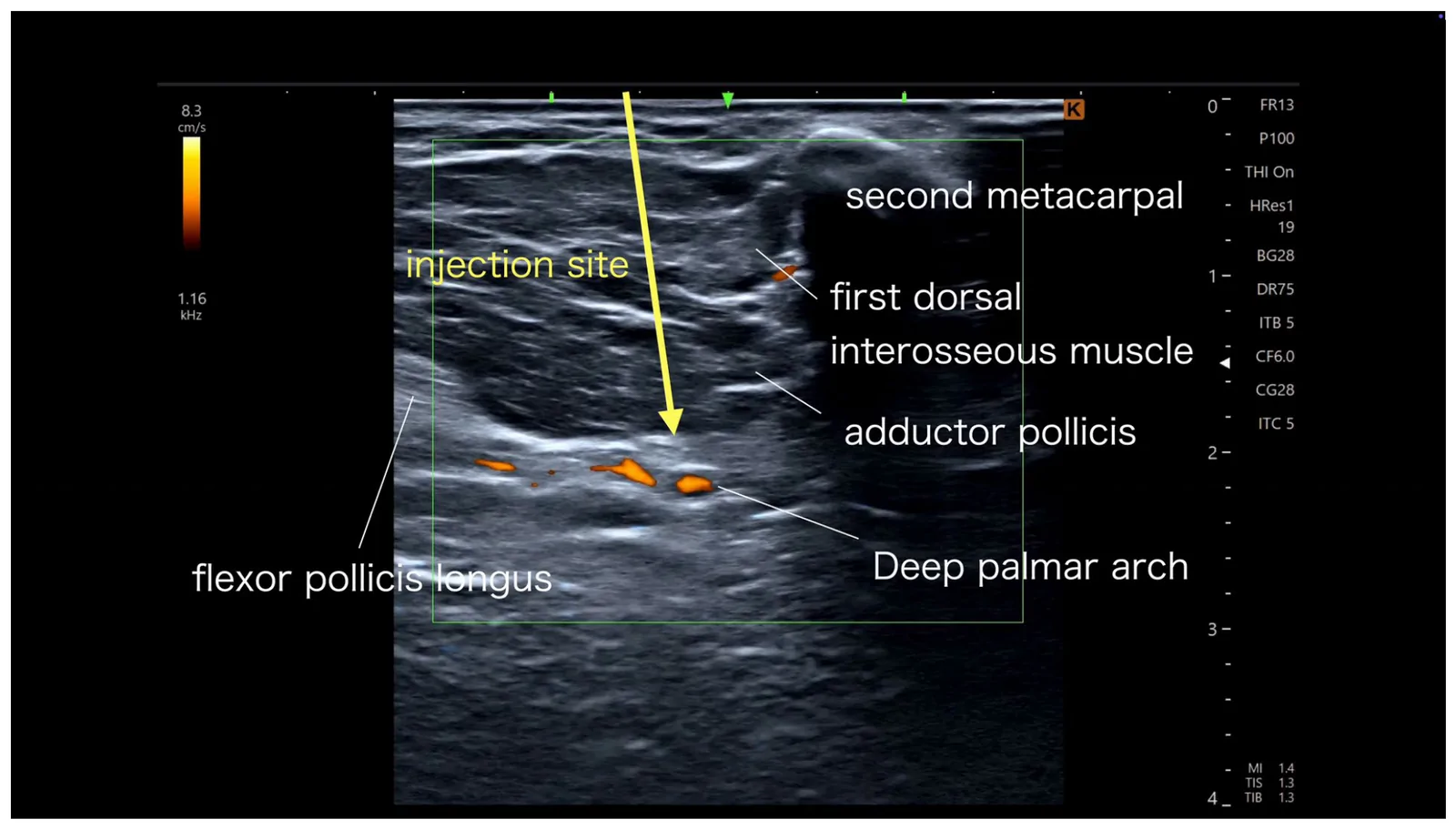
Point 7—first dorsal interosseous/adductor pollicis/deep palmar arch: ultrasound-guided fascia hydrorelease. The green rectangle indicates the color Doppler region of interest, within which the orange signal corresponds to flow in the deep palmar arch. The yellow arrow indicates the needle trajectory toward the injection site.

Procedure (Video S7):

(1) Position the dorsum facing up in forearm pronation; the operator works from the affected side;
(2) Place the probe between the first and second metacarpals;
(3) While scanning, passively abduct the patient’s thumb radially to confirm the gliding of the adductor pollicis and identify its position;
(4) Insert the needle where intermetacarpal tenderness is present and fascial stacking between the first dorsal interosseous and adductor pollicis is confirmed;
(5) Perform US-FHR between the first dorsal interosseous and adductor pollicis, between the adductor pollicis and FPL, and between the adductor pollicis and the deep palmar arch (Figure 11).

#### 3.2.8. Point 8: Adductor Pollicis

The adductor pollicis muscle, with its oblique and transverse heads, is a major contributor to thumb adduction force. Fascial abnormalities of this muscle are often overlooked in conventional evaluation.

**Anatomy:** Adductor pollicis (oblique head and transverse head).

**Indication:** Pain on thumb adduction; grip weakness.

Pressure pain sites: Palmar side from ulnar aspect of proximal phalanx base to third metacarpal.

Pain reproduction movements: (1) Thumb—ulnar adduction resistance and palmar adduction resistance; (2) index finger—abduction resistance and MCP joint resistance with IP joint in extension.

**Figure 12:**
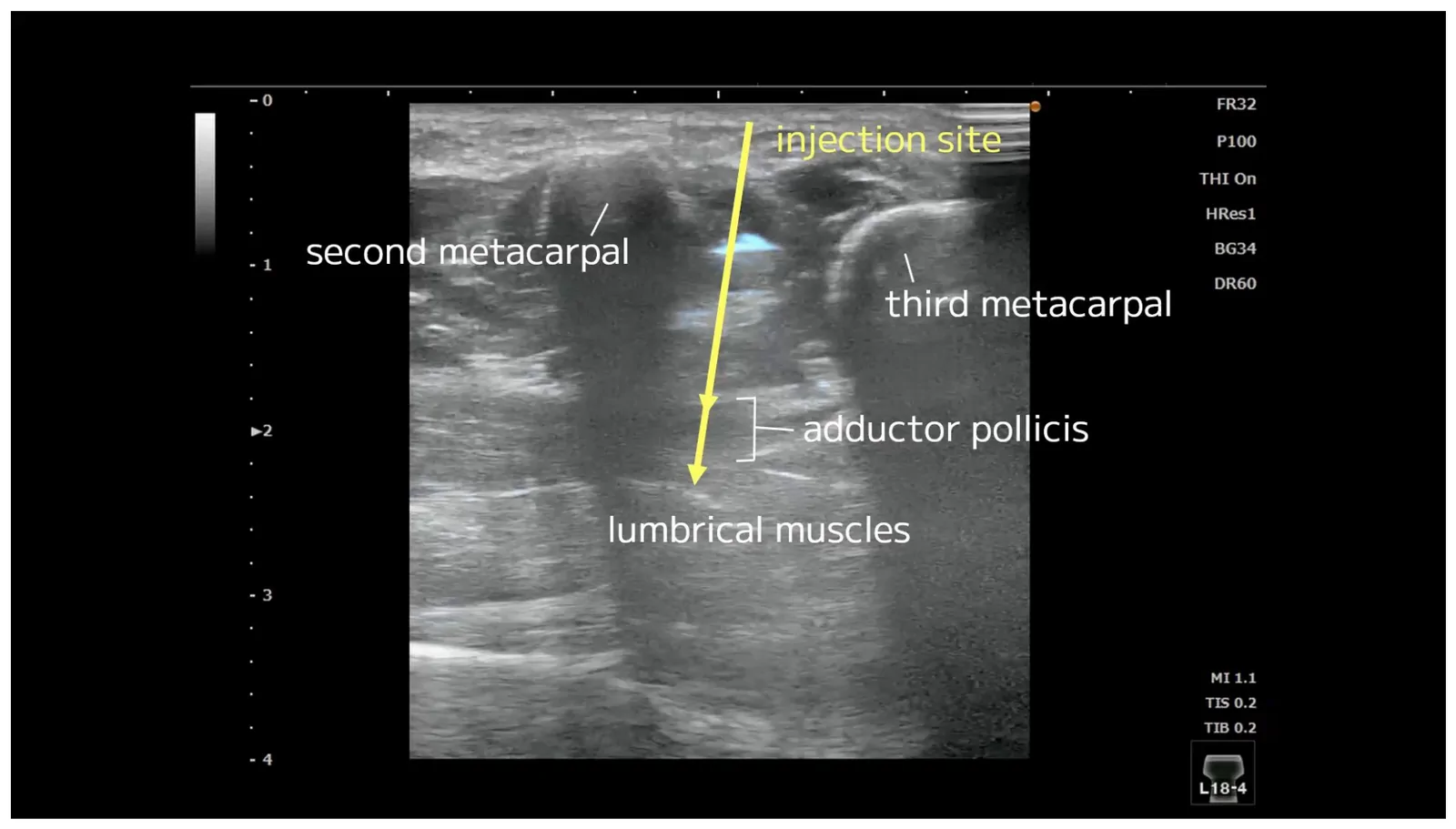
Point 8—adductor pollicis (oblique + transverse heads): ultrasound-guided fascia hydrorelease; yellow arrow indicates the injection site.

Procedure (Video S8):

(1) Position the dorsum facing up in forearm pronation;
(2) Place the probe between the second and third metacarpals;
(3) While scanning, passively abduct the patient’s thumb to confirm the gliding of the adductor pollicis and identify its position;
(4) Insert the needle where intermetacarpal tenderness is present and fascial stacking is confirmed;
(5) Perform US-FHR between the dorsal interosseous and adductor pollicis, and between the adductor pollicis and lumbricals (typically 2 locations) (Figure 12).

#### 3.2.9. Point 9: Deep Palmar Arch/Dorsal and Palmar Interossei

**Figure 13:**
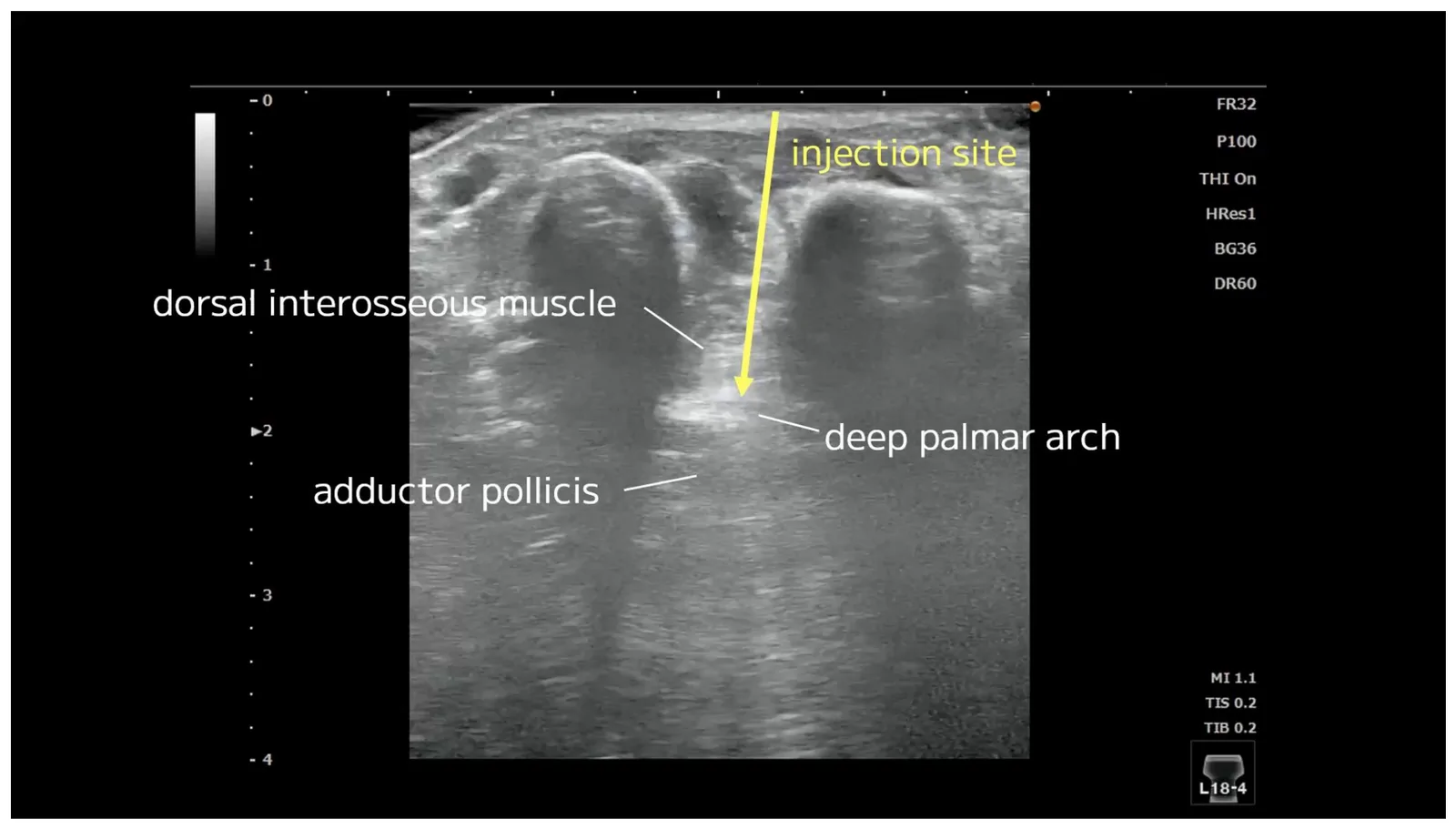
Point 9—deep palmar arch/dorsal and palmar interossei: ultrasound-guided fascia hydrorelease; yellow arrow indicates the injection site.

The deep palmar arch (deep branch of the radial artery) runs between the dorsal and palmar interossei. Fascial adhesion in this deep plane can contribute to deep hand pain and impaired finger coordination. The dorsal approach to the deep palmar arch (Point 9) is illustrated in Figure 13.

**Anatomy:** Dorsal interossei, palmar interossei, and deep palmar arch.

**Indication:** Deep hand pain and grip dysfunction.

Pressure pain sites: Artery itself (between metacarpals).

Pain reproduction movements: Often difficult to reproduce pain with specific movements.

Orthopedic tests: Various orthopedic tests negative.

Ultrasound assessment: Doppler response of deep palmar arch shows side-to-side asymmetry compared with the unaffected side, or compression of the arterial wall is observed.

Procedure (Video S9):

(1) Position the dorsum facing up in forearm pronation;
(2) Place the probe between the metacarpals;
(3) While scanning, passively flex and extend the patient’s fingers to observe the gliding of the dorsal and palmar interossei and to identify the plane of the deep palmar arch;
(4) Use the Doppler mode to confirm pulsation of the deep palmar arch;
(5) Insert the needle where intermetacarpal tenderness is present and fascial stacking is confirmed;
(6) Perform US-FHR between the dorsal interosseous muscles and the deep palmar arch.

### 3.3. Palmar Approach Releases

#### 3.3.1. Point 10: Thenar Muscles (Abductor Pollicis Brevis/Opponens Pollicis/Flexor Pollicis Brevis/Metacarpal)

The thenar muscles form superficial and deep layers over the first metacarpal. Fascial densification between these layers restricts thumb mobility and contributes to pain.

- **Superficial layer:** Abductor pollicis brevis and opponens pollicis.
- **Deep layer:** Flexor pollicis brevis (superficial and deep heads); flexor pollicis longus.
- **Deepest:** Adductor pollicis.

**Indication:** Thumb pain conditions, restricted thumb opposition, and wrist motion pain.

Pressure pain sites: Thenar muscles.

Pain reproduction movements: (1) Thumb—palmar abduction resistance, (2) CMC joint—internal rotation + flexion + abduction resistance, and (3) MCP joint—flexion resistance.

**Figure 14:**
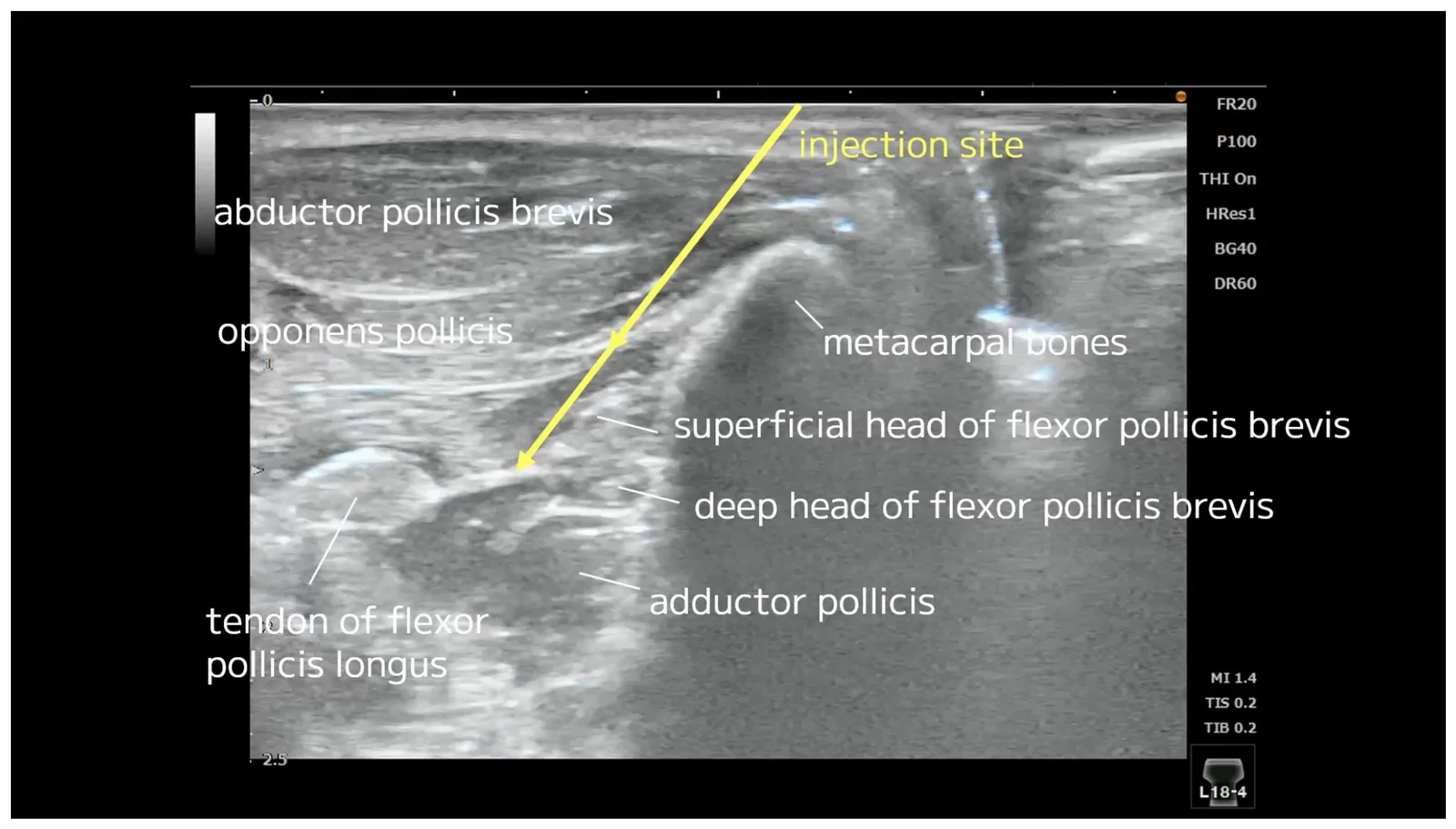
Point 10—thenar muscles: ultrasound-guided fascia hydrorelease; yellow arrow indicates the injection site.

Procedure (Video S10):

(1) Position the palm facing up; the operator works from the affected side;
(2) Place the probe over the thenar eminence;
(3) Use the FPL tendon (visible as a hyperechoic foot-shaped structure) as a landmark to identify the APB, opponens pollicis, FPB, and adductor pollicis;
(4) Insert the needle where tenderness is present and fascial stacking is confirmed;
(5) Perform US-FHR between the APB, opponens pollicis, FPL, FPB, and adductor pollicis at multiple sites (typically 3 locations) (Figure 14).

#### 3.3.2. Point 11: Median Nerve/Transverse Carpal Ligament

Direct fascial plane release within the carpal tunnel. Saline injection between the transverse carpal ligament and the median nerve may reduce neural compression [30]. This point is the primary treatment site for carpal tunnel syndrome.

**Anatomy:** Transverse carpal ligament; median nerve.

**Indication:** Carpal tunnel syndrome; median nerve entrapment.

Inspection: Thenar muscle atrophy; ape hand deformity.

Pressure pain sites: Median nerve (directly over transverse carpal ligament).

Orthopedic tests: (1) Tinel sign positive, (2) Phalen test positive, and (3) thenar muscle weakness.

Neurological assessment: At the carpal tunnel level, sensation differs between the medial and lateral sides of the fourth digit.

**Figure 15:**
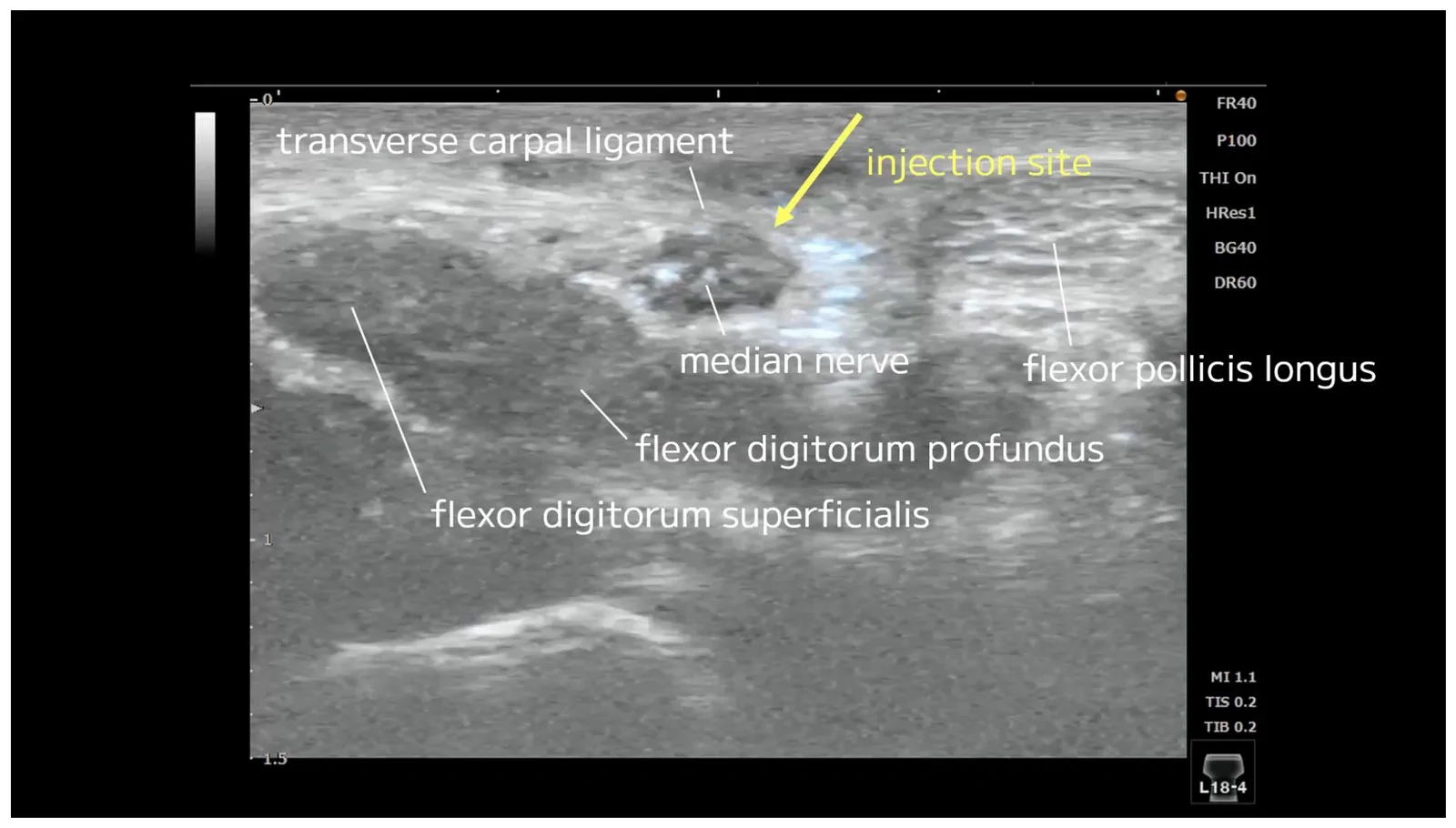
Point 11—median nerve/transverse carpal ligament: ultrasound-guided fascia hydrorelease; yellow arrow indicates the injection site.

Procedure (Video S11):

(1) Position the forearm in supination with the palm facing up;
(2) Place the probe directly over the transverse carpal ligament;
(3) Identify the median nerve deep to the transverse carpal ligament;
(4) Confirm fascial stacking and perform US-FHR between the transverse carpal ligament and median nerve;
(5) If symptoms do not improve, gradually advance the needle while injecting fluid to release the fascia between the median nerve and surrounding structures (Figure 15).

#### 3.3.3. Point 12: Median Nerve (Paraneural Sheath and Interfascicular Epineurium)

Release of the paraneural sheath (paraneurium) and interfascicular epineurium of the median nerve. After releasing between the transverse carpal ligament and median nerve at Point 11, saline is injected into the paraneural sheath and interfascicular epineurium to further reduce intraneural compression. This releases adhesions between nerve fascicles and is intended to restore nerve mobility.

**Anatomy:** Paraneural sheath of the median nerve; interfascicular epineurium.

**Indication:** Carpal tunnel syndrome (when Point 11 provides insufficient improvement); internal entrapment of the median nerve.

Pressure pain sites: Median nerve (directly beneath the transverse carpal ligament).

Pain reproduction movements: The same as Point 11.

**Figure 16:**
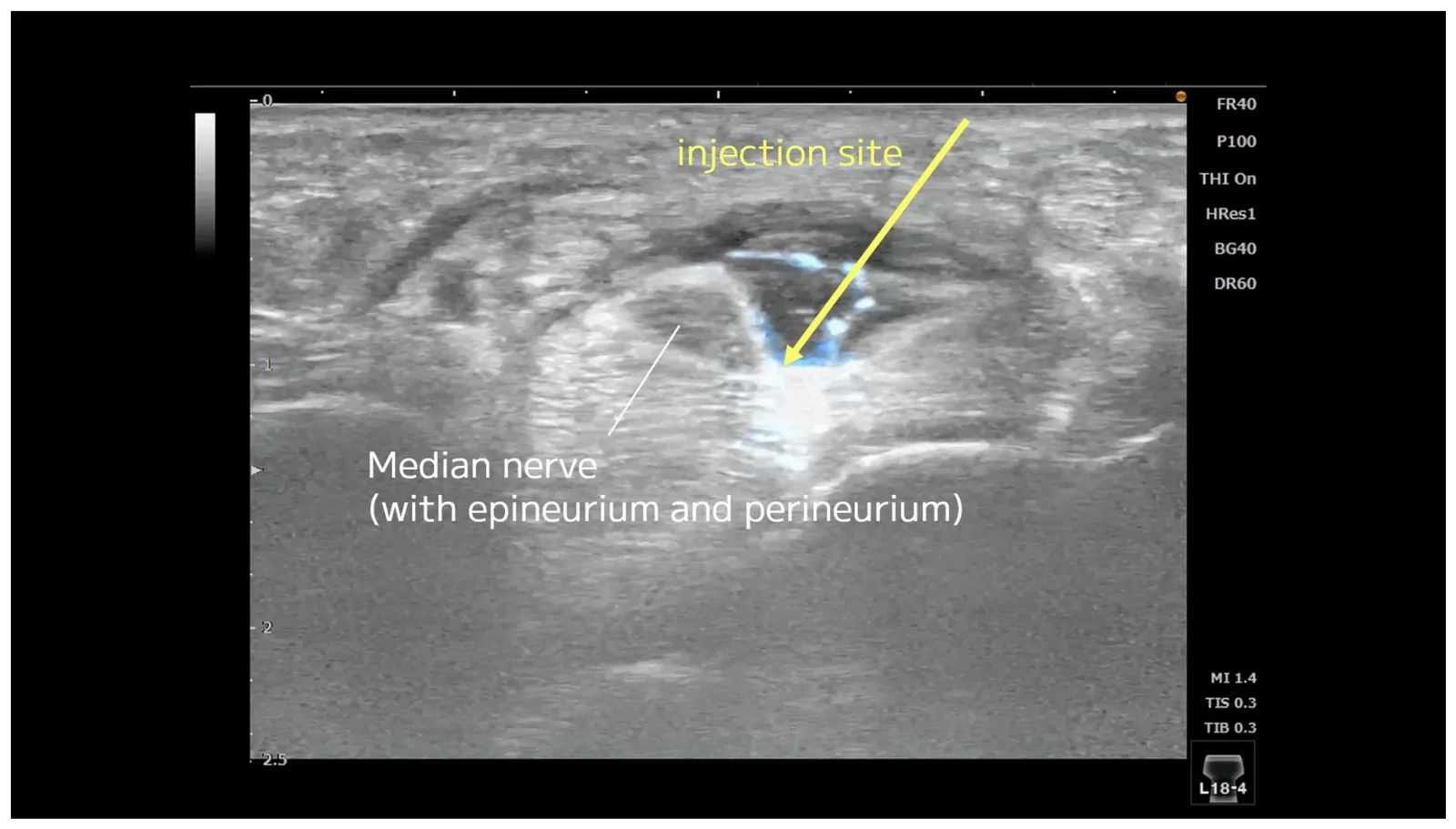
Point 12—median nerve–paraneural sheath/interfascicular epineurium: ultrasound-guided fascia hydrorelease; yellow arrow indicates the injection site.

Procedure (Video S12):

(1) Position as in Point 11;
(2) Perform this procedure when symptoms persist after the transverse carpal ligament-median nerve release (Point 11);
(3) Gradually advance the needle while injecting fluid to release the paraneural sheath of the median nerve;
(4) Continue advancing to release the interfascicular epineurium (US-FHR);
(5) Inject saline between nerve fascicles to release interfascicular adhesions (Figure 16).

#### 3.3.4. Point 13: Median Nerve/FPL/FCR Interface

The median nerve lies between the flexor carpi radialis (FCR) and flexor pollicis longus (FPL), adjacent to the radial artery. Fascial densification in this region can compress the median nerve before it enters the carpal tunnel.

**Anatomy:** Median nerve, FCR, FPL, and radial artery.

**Indication:** Carpal tunnel syndrome, forearm pain, and thumb pain conditions.

Pressure pain sites: (1) Transverse carpal ligament (over median nerve, FCR tendon, FPL tendon, and radial artery), (2) FCR muscle belly, and (3) FPL muscle belly.

Pain reproduction movements: (1) Wrist—radial deviation + palmar flexion resistance (resistance on palmar surface of index/middle metacarpals); (2) thumb—IP joint flexion resistance.

Orthopedic tests: (1) Nerve tension test—median nerve test positive; (2) Tinel sign positive.

**Figure 17:**
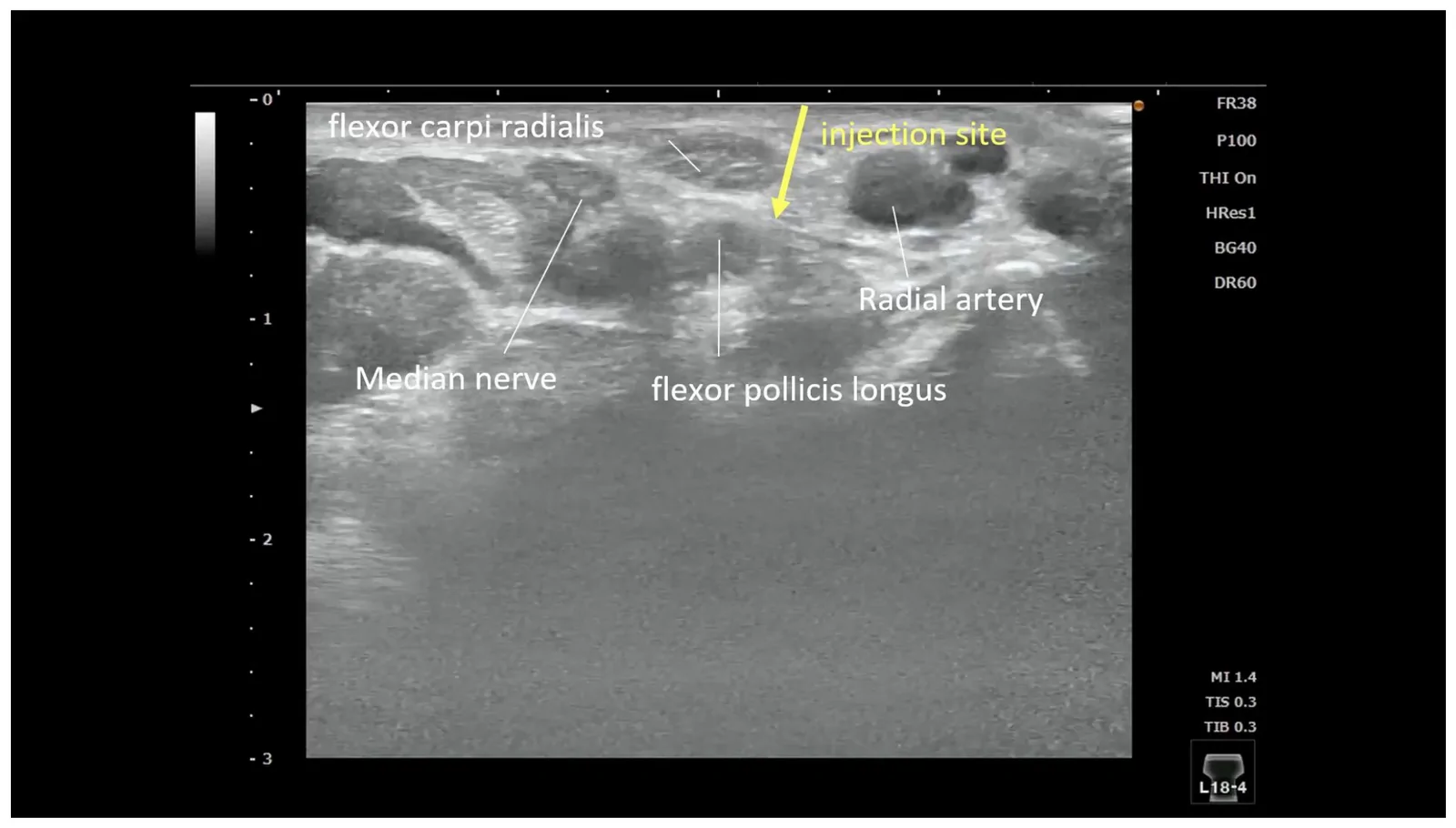
Point 13—median nerve/FPL/FCR interface: ultrasound-guided fascia hydrorelease; yellow arrow indicates the injection site.

Procedure (Video S13):

(1) Position the forearm in supination with the palm facing up; the operator works from the affected side;
(2) Place the probe over the distal radioulnar joint space;
(3) Identify the adjacent median nerve, FPL, FCR, and radial artery;
(4) Insert the needle where tenderness is present and fascial stacking is confirmed;
(5) Perform US-FHR between the median nerve, FPL, FCR, and radial artery (Figure 17).

#### 3.3.5. Point 14: Median Nerve/Flexor Pollicis Longus (Carpal Tunnel)

Deep to the transverse carpal ligament, the median nerve and flexor pollicis longus (FPL) are adjacent. Fascial densification in this area contributes to carpal tunnel syndrome and thumb pain.

**Anatomy:** Transverse carpal ligament, median nerve, and flexor pollicis longus.

**Indication:** Carpal tunnel syndrome and thumb pain conditions.

History: Numbness or pain in the thumb.

Pressure pain sites: FPL tendon (base of the metacarpal).

Pain reproduction movements: Thumb—IP joint flexion resistance.

**Figure 18:**
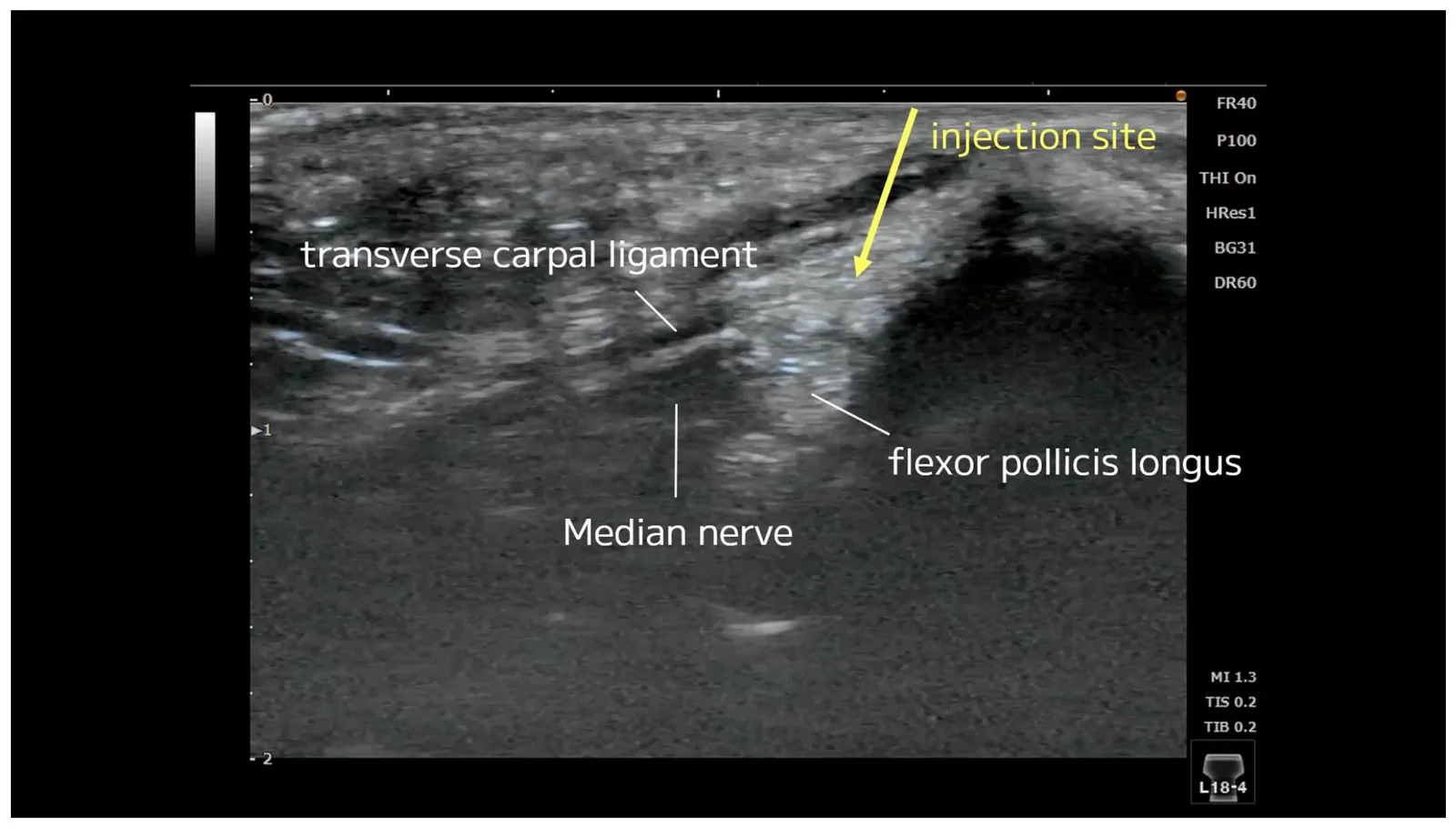
Point 14—median nerve/flexor pollicis longus (carpal tunnel): ultrasound-guided fascia hydrorelease; yellow arrow indicates the injection site.

Procedure (Video S14):

(1) Position the forearm in supination with the palm facing up;
(2) Place the probe directly over the transverse carpal ligament and confirm the median nerve position;
(3) Slide the probe radially to identify the site where the median nerve and FPL tendon are adjacent;
(4) Insert the needle where tenderness is present and fascial stacking is confirmed;
(5) Perform US-FHR between the median nerve and FPL tendon (Figure 18).

#### 3.3.6. Point 15: Palmar Radiocarpal and Palmar Intercarpal Ligaments/Median Nerve

In this concept, the transverse carpal ligament (Point 11) is regarded as the superficial palmar band over the tunnel, whereas the palmar radiocarpal and palmar intercarpal ligaments [28,29] may represent a deeper palmar ligamentous layer adjacent to the carpal bones that has been proposed to contribute to the deep palmar boundary of the carpal tunnel. This complex frequently develops internal densification (stacking fascia) that impairs its normal gliding and contributes to wrist/thumb pain symptoms. Ultrasound-guided FHR is applied directly into this ligament complex, targeting intra-ligamentous densified layers (stacking fascia within the ligaments), thereby releasing the densified collagen sublayers and restoring physiological gliding of the structure (see Video S15). Combined release of both structures (Points 11 and 15) is intended to achieve more complete decompression of the carpal tunnel.

**Anatomy:** Palmar radiocarpal and palmar intercarpal ligaments [28,29]; median nerve.

**Indication:** Carpal tunnel syndrome (adjunct).

Pressure pain sites: Directly over the palmar radiocarpal and palmar intercarpal ligaments (deep to the transverse carpal ligament).

Pain reproduction movements: Wrist—palmar flexion resistance.

**Figure 19:**
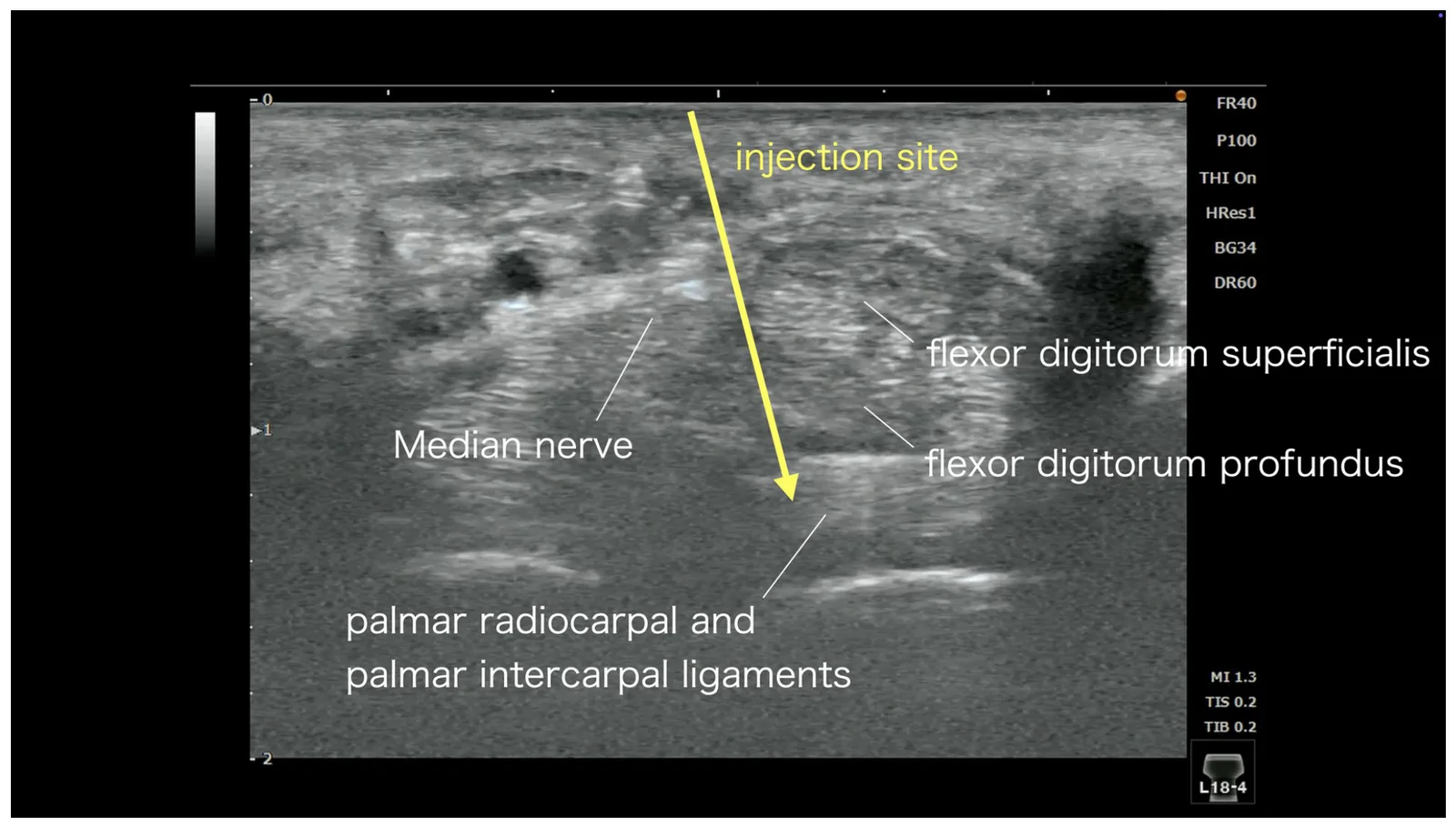
Point 15—palmar radiocarpal and palmar intercarpal ligaments/median nerve: ultrasound-guided fascia hydrorelease; yellow arrow indicates the injection site.

Procedure (Video S15):

(1) Position the forearm in supination with the palm facing up;
(2) Place the probe directly over the palmar radiocarpal and palmar intercarpal ligaments;
(3) Identify the palmar radiocarpal and palmar intercarpal ligaments (deep to the transverse carpal ligament) and its relationship with the median nerve;
(4) Insert the needle where tenderness is present and fascial stacking is confirmed;
(5) Perform US-FHR into the palmar radiocarpal and palmar intercarpal ligaments, targeting intra-ligamentous densified layers (stacking fascia) (Figure 19).

### 3.4. Pathology-Specific Application of the 15-Point Protocol

#### 3.4.1. de Quervain’s Tenosynovitis

Traditional treatment includes splinting, NSAIDs, and corticosteroid injection into the first-extensor-compartment sheath. Surgical tendon sheath release is reserved for refractory cases. FHR addresses not only the first-compartment sheath but also the surrounding fascial continuum—Points 1 and 5 directly, plus the adjacent dorsal structures targeted at Points 2–4—which conventional treatment may not fully address. In the authors’ clinical experience, some patients who do not achieve satisfactory relief with isolated first-compartment injection appear to respond to the broader fascial approach; this pattern has not yet been quantified in controlled studies.

#### 3.4.2. Intersection Syndrome

Intersection syndrome results from friction at the crossing of the first and second extensor compartments. While conventional treatment focuses on the intersection point itself, this approach recognizes that the fascial abnormality extends both proximally and distally. Points 3 and 4 directly target the two intersection zones.

#### 3.4.3. CMC Osteoarthritis

CMC osteoarthritis has traditionally been attributed to cartilage degeneration at the trapeziometacarpal joint. However, the fascial perspective suggests that when fascial densification develops in both the extensor (Points 2–4) and flexor (Points 7–8, 10) compartments surrounding the CMC joint, abnormal mechanical loading may be imposed on the joint during both extension and flexion through epimuscular myofascial force transmission [31]. Since CMC osteoarthritis is often accompanied by joint instability, direct capsular release may exacerbate this instability. Therefore, the FHR approach targets the surrounding extensor fascia (Points 2–4) and flexor fascia (Points 7–8, 10) rather than the joint capsule itself, thereby indirectly reducing abnormal mechanical loading on the joint. Beyond mechanical unloading, there is growing recognition that vascular pathology plays a role in the initiation and progression of osteoarthritis. Findlay proposed that episodically reduced blood flow through small vessels in subchondral bone, caused by venous stasis or microemboli, contributes to ischemia-driven cartilage degradation and osteocyte apoptosis [32]. Conaghan further hypothesized that progressive OA may represent an atheromatous vascular disease of subchondral bone [33], and recent evidence suggests that subchondral blood flow abnormalities may precede cartilage degeneration as an early pathological event [34]. In this context, periarterial fascial release at Point 6 (anatomical snuffbox) (technique conceptually analogous to periarterial FHR reported in a different anatomical region—the cervical periarterial region—in a related prospective interventional study by our group [35]) acquires particular significance for CMC osteoarthritis. The radial artery traverses the snuffbox before entering the deep palm and giving rise to the princeps pollicis artery, which is the principal blood supply to the thumb and CMC joint region. Fascial entrapment at this vascular–tendon crossing point—where the radial artery crosses deep to the EPL tendon and the ECRL/ECRB tendons—may compromise arterial flow to the CMC joint. Release of this fascial entrapment through FHR may, therefore, enhance periarticular tissue perfusion and metabolism, potentially addressing not only pain but also the vascular component of OA pathophysiology. Additionally, corticosteroid injection is indicated for intra-articular inflammatory pathology, and dextrose prolotherapy may serve as a viable option for symptomatic pain relief and functional improvement [36]. This multilayered approach—mechanical unloading through surrounding fascial release, vascular improvement through periarterial release, and targeted intra-articular treatment—may offer complementary benefits alongside local joint therapy and may reduce pain in some patients even in the presence of significant radiographic degeneration, although controlled outcome data are lacking. Because the radial artery is the common parent vessel supplying this region, periarterial fascial constriction at the more proximal radial artery/first-compartment crossing (Point 5) may likewise compromise downstream perfusion of the trapeziometacarpal joint; release at both radial–artery crossings (Points 5 and 6) is, therefore, proposed to address periarterial constriction affecting CMC subchondral perfusion.

#### 3.4.4. Carpal Tunnel Syndrome

CTS is the most common entrapment neuropathy. While surgical carpal tunnel release remains the gold standard for severe cases, FHR offers a non-surgical alternative for mild-to-moderate CTS. Notably, Evers et al. reported that ultrasound-guided hydrodissection reduces gliding resistance of the median nerve within the carpal tunnel [30]. Points 11, 12, 13, and 14 provide systematic decompression of the median nerve at multiple levels: proximal to, within, and superficial to the carpal tunnel. The advantage over conventional corticosteroid injection is the avoidance of steroid-related tendon weakening and the ability to address the fascial pathology at multiple levels simultaneously.

#### 3.4.5. Myofascial Pain Syndrome (Referred Pain from Proximal Structures)

**Figure 20:**
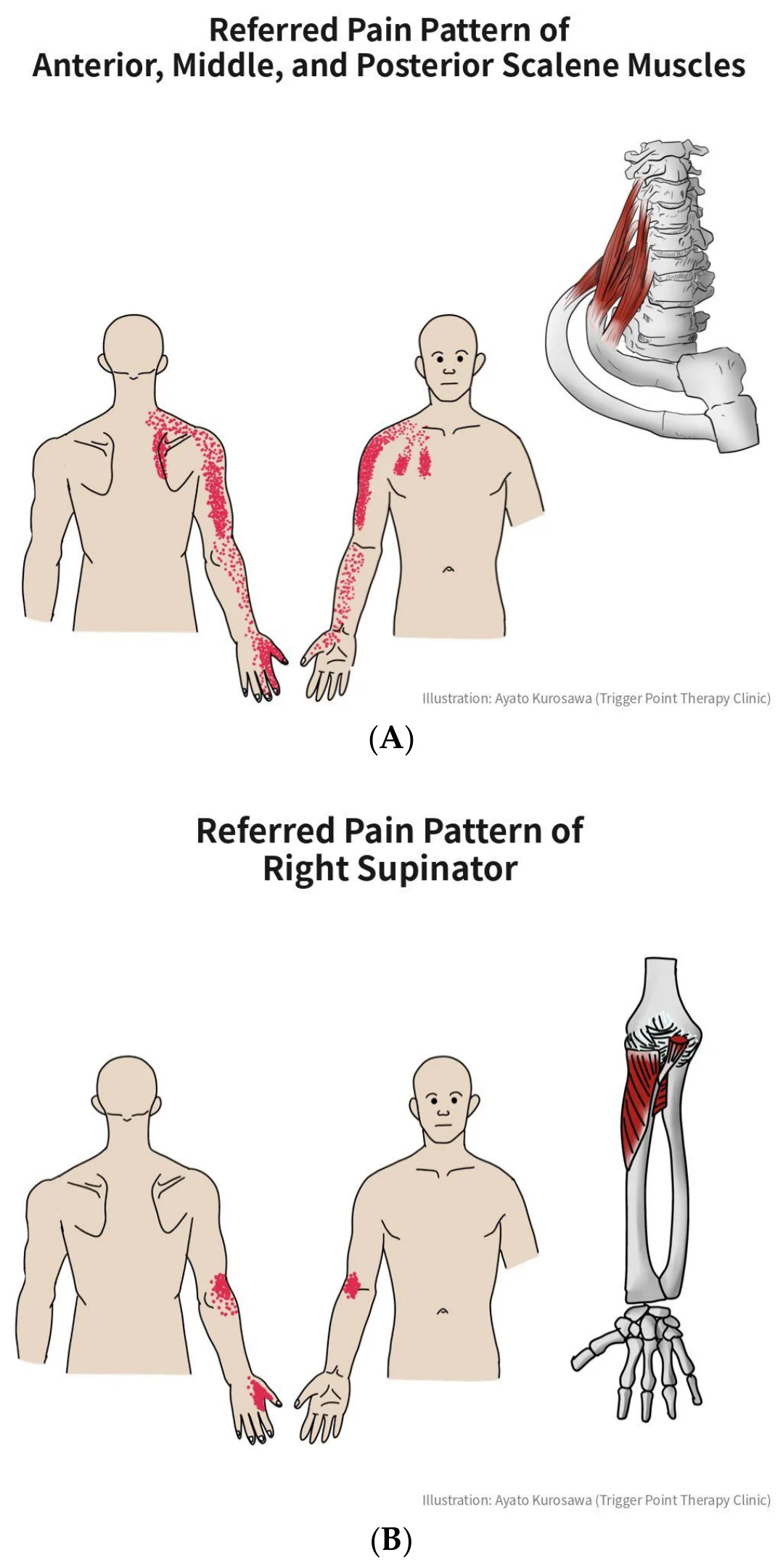

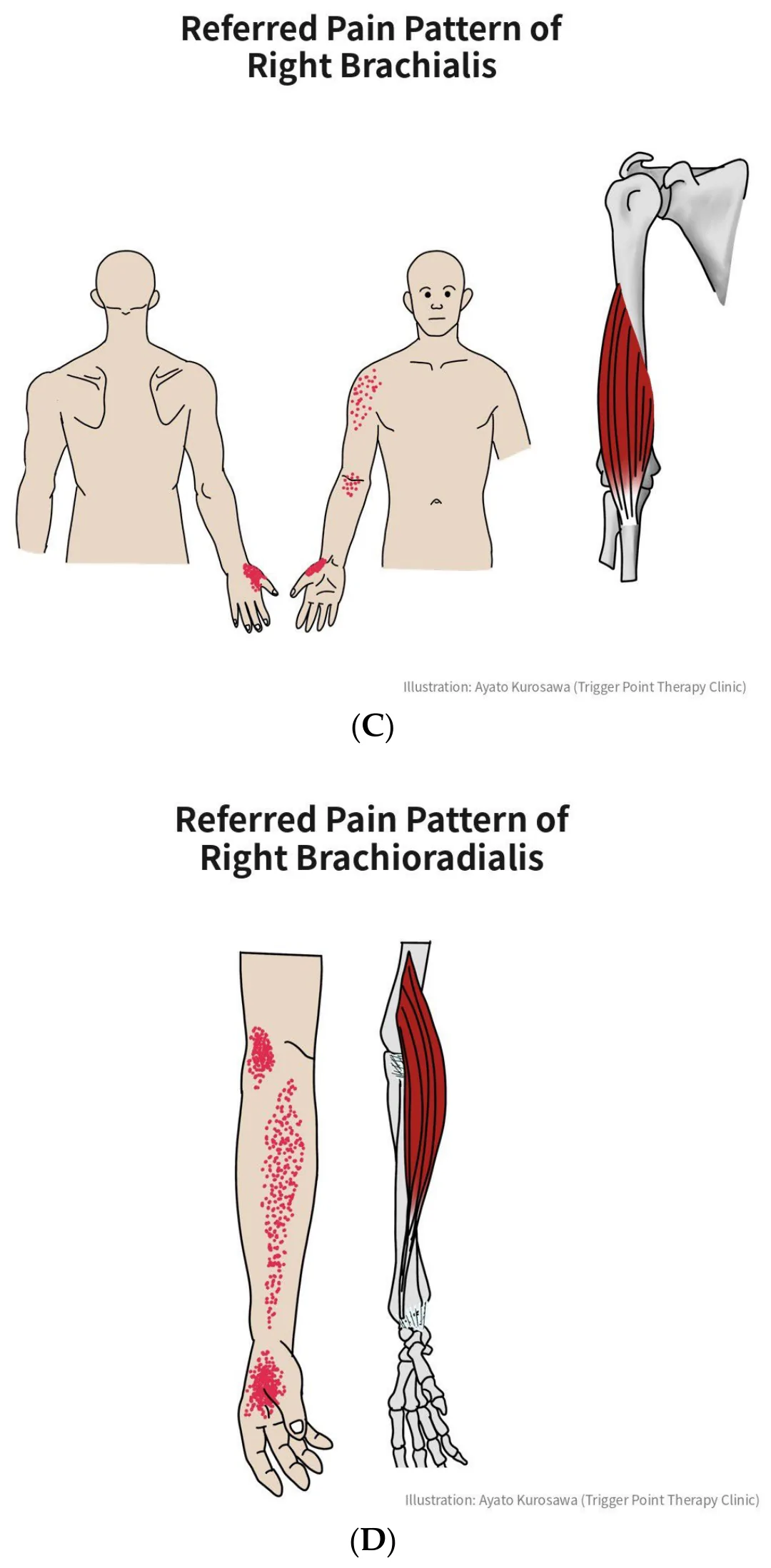
Referred pain patterns from proximal fascial densification to the thumb region. (**A**) Anterior, middle, and posterior scalene muscles: a widespread referred pain area (red stippled region) extending from the shoulder through the upper arm and forearm to the thumb. (**B**) Supinator: referred pain pattern from the lateral elbow to the dorsum of the hand and thumb base. (**C**) Brachialis: referred pain pattern centered on the palmar aspect of the thumb. (**D**) Brachioradialis: referred pain pattern from the lateral forearm to the dorsum of the hand and thumb. These proximal fascial sources may contribute to persistent pain when the local 15-point protocol yields insufficient improvement. The referred pain distributions shown are original illustrations created with reference to the clinical descriptions in the *Trigger Point Manual* [25]. Illustration: Ayato Kurosawa (Trigger Point Therapy Clinic).

The fascial continuum extends from the cervicoscapular region through the upper arm and forearm to the hand. Fascial densification of the scalene muscles (anterior, middle, and posterior), brachialis, brachioradialis, and supinator muscles can elicit referred pain extending to the thumb region, consistent with the classical trigger point referral patterns documented for these muscles [25]. Specifically, fascial densification of the anterior, middle, and posterior scalene muscles produces a widespread referred pain pattern extending from the shoulder through the upper arm and forearm to the thumb (Figure 20A); fascial densification of the supinator generates referred pain from the lateral elbow to the dorsum of the hand and thumb base (Figure 20B); and fascial densification of the brachialis characteristically refers pain to the palmar aspect of the thumb (Figure 20C). These referred pain patterns may explain the “spreading pain from the thumb to the forearm” commonly reported by patients with these conditions. When the local 15-point protocol does not achieve sufficient improvement, evaluation and treatment of these proximal fascial sources may be necessary for complete symptom resolution.

## 4. Discussion

This clinical protocol article synthesizes anatomical, mechanistic, and clinical-experience-based observations, suggesting that the five common thumb and wrist conditions—de Quervain’s tenosynovitis, CMC osteoarthritis, carpal tunnel syndrome, intersection syndrome, and myofascial pain syndrome—share a common fascial substrate characterized by stacking fascia on ultrasound examination. This shared pathological finding, together with the anatomical continuity of fascial planes from the thumb–wrist region into the forearm, supports a unified understanding of thumb pain. The clinical observation that multiple pain sources may coexist within a connected fascial network has led the authors to informally consider the possibility of a broader unifying framework; this possibility, tentatively referred to by the authors as “Thumb Pain Syndrome” (TPS), is presented here as a hypothesis for future investigation rather than a defined clinical entity, and requires prospective validation before it can be considered an established framework, consistent with the cautious framing adopted in earlier Japanese-language reports [5].

Recent biomechanical evidence from cadaveric studies has suggested that hydrorelease significantly reduces the gliding resistance force between fascial layers [27], providing supportive evidence for the proposed mechanical mechanism underlying FHR. Furthermore, the fascial perspective on pain is gaining broader recognition, with growing reports suggesting potential involvement of fascial abnormalities in conditions such as complex regional pain syndrome [37]. Moreover, an integrative model has been proposed that positions fascial densification and impaired gliding as important peripheral drivers of chronic pain in myofascial pain syndrome [38].

The fascial perspective on thumb pain offers several clinical advantages:

**Comprehensive assessment:** The four-direction screening test (flexion, extension, abduction, and adduction) with three modes (contraction, resistance, and stretch) provides a systematic method for identifying the specific fascial structures involved in each patient’s presentation.

**Diagnostic significance:** Conventional clinical tests such as the Finkelstein test, Phalen test, and Grind test have traditionally been used to diagnose specific individual conditions (de Quervain’s tenosynovitis, carpal tunnel syndrome, and CMC osteoarthritis, respectively). However, from the perspective of shared fascial abnormalities across these five conditions, the simultaneous positivity of multiple tests may constitute supportive clinical observation of the presence of fascial densification as a common pathological substrate. This “cross-test positivity” also provides theoretical support for the comprehensive treatment approach of the 15-point protocol.

**Targeted multi-point treatment:** Rather than treating a single diagnosis, the 15-point protocol addresses the entire fascial network of the thumb–wrist region. The specific points treated in each patient are determined by the screening examination findings and ultrasound evaluation.

**Molecular rationale:** The pathophysiology of fascial densification involves multiple molecular pathways. The gliding property of fascial layers is caused by hyaluronic acid (HA) within the loose connective tissue between adjacent fascial planes [10,39]. Under pathological conditions, HA aggregates through supramolecular interactions, increasing viscosity and transforming the normally lubricating layer into a viscous adhesive substance that impairs fascial gliding [39,40] and may contribute to nociceptive signaling through the sensory innervation of fascia [41,42]. Beyond HA aggregation, fascial densification involves broader changes in the extracellular matrix (ECM), including increased collagen deposition, upregulation of MMPs/TIMPs, fibroblast-to-myofibroblast transformation, and increased TGF-β expression, creating a self-reinforcing cycle of fascial stiffening and pain [43]. Recent research has highlighted the role of the YAP/TAZ pathway, which functions as a central mechanosensor in fibroblasts [44,45,46]. In densified fascia, increased tissue stiffness may activate YAP/TAZ nuclear translocation, potentially promoting further ECM production and myofibroblast differentiation, thereby creating a mechanobiological positive feedback loop [47]. We have proposed the “Fascial Memory Reset Hypothesis,” suggesting that FHR may break this feedback loop by acutely reducing mechanical tension at fascial planes, potentially leading to YAP/TAZ cytoplasmic sequestration and eventual normalization of ECM turnover [26]. However, this hypothesis remains a theoretical framework requiring further experimental validation. The therapeutic mechanism of FHR likely involves multiple pathways: mechanical separation of densified layers [27], HA dilution reducing viscosity [39,40], mechanotransduction reset via YAP/TAZ inactivation, nerve decompression through fascial plane separation, and vascular improvement through restored fascial mobility, although the relative contribution of each pathway remains to be elucidated.

**Controlled trial support for the myofascial component:** Interfascial injection (fascia hydrorelease) has demonstrated efficacy for myofascial pain syndrome in prospective, randomized, double-blinded trials [48], providing controlled trial support for the myofascial pain component addressed by the present protocol; however, the thumb-specific fifteen-point protocol itself has not yet been evaluated in a controlled trial. Minimally invasive: FHR using normal saline avoids the risks associated with corticosteroid injection (tendon weakening and skin atrophy) and surgical intervention (scarring and recovery time).

**Integrated therapeutic framework:** The proposed US-FHR approach for these five converging conditions is characterized by its integration of three therapeutic axes: (1) potential vascular improvement through periarterial fascial release to restore local circulation, (2) mechanical unloading through extensor and flexor fascial release to reduce abnormal force transmission, and (3) range of motion restoration through fascial release to recover fascial gliding. For refractory cases where conventional pharmacological approaches such as peripheral nerve blocks and corticosteroid injections have reached their limits, this approach may offer a new paradigm focusing on fascial biomechanics and mechanotransduction (non-pharmacological hydrorelease). While normalization of mechanotransduction as proposed in the fascial memory reset hypothesis [26] requires further validation, this framework theoretically suggests that the approach may simultaneously address multiple pathological mechanisms.

### 4.1. Levels of Evidence

For transparency, the concepts and claims discussed throughout this article can be organized into three evidentiary tiers. Tier 1 (established findings supported by the existing literature) includes standard anatomical descriptions of the thumb–wrist–forearm complex, the biochemistry of hyaluronic-acid-related fascial densification and impaired interlayer gliding, the general principles of ultrasound-guided injection technique, epidemiological data on the five converging thumb pain conditions, the reduction in gliding resistance between fascial layers following hydrorelease reported in cadaveric studies, and the demonstration of the efficacy of interfascial injection (fascia hydrorelease) for myofascial pain syndrome in prospective, randomized, double-blinded trials [48]. Tier 2 (plausible but not yet clinically validated mechanistic hypotheses) includes the proposal that multi-layered hyperechoic bands with reduced interlayer gliding correspond to “stacking fascia,” a previously described sonographic sign of fascial densification (a hyperechoic, stripe-shaped lesion) [12,48], proposed to be clinically meaningful; the proposal that the fifteen-point protocol described here constitutes an integrated treatment framework for five converging thumb pain conditions; the proposal that periarterial release around the radial artery may contribute to subchondral vascular improvement in CMC osteoarthritis; and the broader informal framing of these conditions as an interconnected group of fascially driven presentations (“Thumb Pain Syndrome” (TPS)). These proposals are biologically plausible and consistent with existing anatomical and mechanistic evidence, but have not yet been validated by prospective, controlled, or randomized studies. Tier 3 (observations derived from the authors’ clinical experience) includes detailed procedural descriptions, injectate volumes, needle trajectories, tender point patterns, and clinical response patterns described in the POINT-by-POINT sections, which reflect the authors’ long-term clinical experience at Kimura Pain Clinic (approximately 360,000 US-FHR procedures (an approximate internal running count of the primary author’s ultrasound-guided procedures at Kimura Pain Clinic; not derived from a prospective registry, an aggregated outcome database, or an audited clinical dataset; the figure is provided as an order-of-magnitude indication of procedural exposure only) across more than a decade at a single center). These observations have not been aggregated as formal outcome data; they are shared here to inform practical implementation rather than as evidence of efficacy. Readers are invited to interpret each element of the article in light of the tier to which it corresponds.

### 4.2. Comparison with Established Conservative Management

To help readers position ultrasound-guided fascia hydrorelease within current clinical practice, we compare it here with established conservative management strategies for the five thumb-pain conditions covered by this protocol. Rehabilitation and physical/exercise therapy remain first-line management for most thumb pain conditions and address movement patterns, strength, and functional load. The fifteen-point US-FHR protocol proposed here is not intended to replace these modalities but to address a proposed fascial substrate (densification and impaired interlayer gliding) that may persist despite adequate rehabilitation; US-FHR may be considered an adjunct to, or a preparatory intervention before, structured rehabilitation. Corticosteroid injection is widely used for de Quervain’s tenosynovitis, CMC osteoarthritis, and carpal tunnel syndrome, with well-documented short-to-intermediate efficacy and known tissue-related side effects with repeated use. US-FHR targets a mechanistic axis (proposed fascial densification and impaired gliding) that is distinct from the axis targeted by corticosteroid (inflammatory modulation) and uses saline rather than a pharmacologically active agent as the injectate. In this respect, US-FHR may serve as a potentially complementary—rather than a competing option—in patients for whom repeated corticosteroid injection is undesirable or has been exhausted. Surgical release (e.g., first dorsal compartment release for de Quervain’s tenosynovitis, arthroplasty or arthrodesis for CMC osteoarthritis, carpal tunnel release for CTS, or debridement for intersection syndrome) is generally reserved for refractory presentations, or for cases in which structural pathology is established. US-FHR is proposed here as an intermediate, minimally invasive option that may be considered before surgical escalation in appropriately selected patients, particularly when fascial densification is the predominant sonographic finding and when structural pathology has not yet reached the surgical threshold. In patients with established fibrosis or advanced structural pathology, US-FHR alone may be insufficient and combined or alternative strategies should be considered. Direct comparative trials are needed to define the optimal patient selection between these modalities. Table 7 compares the present protocol with established conservative modalities for thumb pain.

### 4.3. Limitations

This work has several important limitations that readers should bear in mind when interpreting its content. First, this article is a clinical protocol organized on the basis of a focused (not systematic) literature review and long-term clinical experience at Kimura Pain Clinic, rather than a systematic review or an original controlled study; the observations described here have not been validated through prospective, controlled, or randomized studies. Second, the proposed mechanistic model—including the pathological significance attributed to “stacking fascia” (a previously described sonographic sign of fascial densification [12,48]), the fifteen-point protocol as a treatment framework, and the periarterial vascular contribution to CMC osteoarthritis—remains hypothetical and requires further biomechanical, imaging, and clinical validation. Third, the clinical experience referenced in this article (approximately 360,000 US-FHR procedures (an approximate internal running count of the primary author’s ultrasound-guided procedures at Kimura Pain Clinic; not derived from a prospective registry, an aggregated outcome database, or an audited clinical dataset; the figure is provided as an order-of-magnitude indication of procedural exposure only) across more than a decade at a single center) provides a substantial procedural basis but does not represent quantitatively analyzed outcome data; formal effectiveness assessment awaits future prospective and comparative studies. Fourth, the informal “Thumb Pain Syndrome” framing that the authors have used clinically is presented here only as a hypothesis for future investigation and should not be interpreted as a defined nosological entity. Fifth, while a double-blinded randomized controlled trial supports the efficacy of interfascial injection (fascia hydrorelease) for myofascial pain syndrome [48], the thumb-specific fifteen-point protocol itself has not yet been evaluated in a controlled trial. Evidence supporting fascia hydrorelease for the specific thumb pain conditions discussed here is currently limited, and the protocol described in this article is based on anatomical rationale, mechanistic considerations, the published literature, and the authors’ extensive clinical experience, rather than on high-level prospective comparative clinical studies. Sixth, in cases where fibrosis has become established, fascia hydrorelease alone may be insufficient, and consideration of additional therapeutic options may be warranted; this may represent an important direction for future clinical investigation. Finally, prospective observational studies, randomized controlled trials, and imaging-based validation studies—including comparison with established conservative management strategies such as rehabilitation, physical and exercise therapy, corticosteroid injection, and surgical release—are needed before this protocol can be considered an established diagnostic or therapeutic framework.

## 5. Conclusions

This clinical protocol article has synthesized anatomical rationale, mechanistic considerations, and clinical-experience-based observations to propose a structured fifteen-point ultrasound-guided fascia hydrorelease (US-FHR) protocol for five converging thumb pain conditions—de Quervain’s tenosynovitis, CMC osteoarthritis, carpal tunnel syndrome, intersection syndrome, and myofascial pain syndrome—which may share common fascial abnormalities including densification and impaired interlayer gliding. The protocol comprises nine POINTs via a dorsal approach and six POINTs via a palmar approach (nine + six = fifteen POINTs in total), each linked to a defined anatomical target with a described procedural concept and major safety considerations. Ultrasound findings characterized by multi-layered hyperechoic bands with reduced interlayer gliding correspond to “stacking fascia,” a previously described sonographic sign of fascial densification (a hyperechoic, stripe-shaped lesion) [12,48], and may assist in identifying candidate treatment sites. Periarterial release at the radial artery crossings (POINTs 5 and 6) is presented as a hypothesis-generating approach to CMC osteoarthritis, in which fascial constriction of periarterial tissue may contribute to subchondral vascular compromise. This mechanistic model, together with the broader clinical framework informally referred to by the authors as “Thumb Pain Syndrome,” is presented as a hypothesis for future investigation rather than a defined clinical entity. The proposed protocol may serve as a practical basis for standardization, education, and broader dissemination of US-FHR in this patient population. Prospective observational studies, randomized controlled trials, and imaging-based validation studies—together with direct comparison against established conservative management strategies (rehabilitation, physical and exercise therapy, corticosteroid injection, and surgery)—are needed to evaluate its clinical effectiveness and to test the underlying mechanistic hypotheses. For clinical implementation, the protocol is intended to be delivered in the outpatient setting using standard ultrasound equipment and 27-gauge needles and presupposes clinician proficiency in musculoskeletal ultrasound-guided procedures.

## Figures and Tables

**Table 1:** Screening test for five thumb pain conditions.

Movement	Contraction	Resistance	Stretch	Primary Fascial Targets
Flexion	Active thumb flexion	Resist thumb flexion	Passive thumb extension	FPL, thenar muscles, and median nerve
Extension	Active thumb extension	Resist thumb extension	Passive thumb flexion	EPL, EPB, APL, and extensor retinaculum
Abduction	Active thumb abduction	Resist thumb abduction	Passive thumb adduction	APL, EPB, and 1st/2nd compartment intersection
Adduction	Active thumb adduction	Resist thumb adduction	Passive thumb abduction	Adductor pollicis, thenar muscles, and deep palmar arch

**Table 2:** Diagnostic accuracy of clinical tests and their significance in five thumb pain conditions.

Clinical Test	Test Maneuver	Target Condition	Sensitivity (%)	Specificity (%)	Clinical Significance
Finkelstein test	Thumb flexed into the palm within a closed fist; the wrist is ulnarly deviated to provoke radial styloid pain.	de Quervain’s	Not well established *	Not well established *	Highly specific but with variable sensitivity in the literature; may also be positive with fascial densification (Points 1–2), even without de Quervain’s [17]
Phalen test	The wrist is held in full flexion for about 60 s to provoke median-nerve paresthesia.	CTS	42–85	54–98	Reflects fascial abnormality within carpal tunnel (Points 11–15) [18]
Tinel sign (wrist)	Percussion over the median nerve at the wrist elicits paresthesia in its distribution.	CTS	45–59	78–90	Indicator of perimedian nerve fascial densification [18]
Grind test	Axial compression with rotation of the first metacarpal on the trapezium provokes pain or crepitus.	CMC OA	30–64	80–100	Periarticular fascia (Points 7–10) may cause false negatives [19,20]
Eichhoff test	The examiner grasps the flexed thumb within the patient’s fist and ulnarly deviates the wrist.	de Quervain’s	89	14	Often confused with Finkelstein. Higher false-positive rate with fascial densification [17,21]
WHAT test	The wrist is held in hyperflexion while the thumb is abducted and extended against resistance.	de Quervain’s	99	29	High sensitivity but low specificity. Useful for screening fascial abnormalities [21]
Lever test	A lever (distraction) force applied to the first metacarpal stresses the CMC joint.	CMC OA	82	81	Higher sensitivity than Grind test. Also applicable to periarticular fascia (Points 7–10) assessment [22]
Durkan test	Direct compression over the carpal tunnel for about 30 s provokes median-nerve paresthesia.	CTS	87	90	Direct carpal tunnel compression. May also be positive with perimedian nerve fascial densification [23]
Tinel sign (dorsal wrist)	Percussion over the superficial radial nerve elicits paresthesia over the dorsal thumb.	Wartenberg syndrome	—	—	Superficial radial nerve entrapment. Important for differential diagnosis with these five conditions [24]

Note: Sensitivity and specificity values are for diagnosing each individual condition. In the presence of fascial abnormalities (particularly stacking fascia), these tests may become positive even without the specific target condition, supporting the concept that fascial densification serves as a common pathological substrate that cross-activates multiple clinical tests in these converging conditions [5,10,25]. * The exact sensitivity and specificity of the Finkelstein test are not well established in the literature; the test is generally considered highly specific but with variable sensitivity.

**Table 3:** Pain sources in five thumb pain conditions classified by movement direction and pain type.

Movement Direction	Contraction Pain (Agonist Structures)	Stretch Pain (Antagonist Structures)
**Adduction**	Adductor pollicis Thenar muscles (OP, APB, FPB, FPL, and AdP) 1st dorsal interosseous/FPL/FPB/AdP	1st compartment (APL and EPB) 1st/2nd compartment intersection (APL, EPB/ECRL, and ECRB)
**Flexion**	Thenar muscles (as above) 1st dorsal interosseous/FPL/FPB/AdP Median nerve/FPL Median nerve/FPL/FCR/radial artery Median nerve (TCL, paraneural sheath, and interfascicular epineurium)	1st compartment (APL and EPB) 3rd compartment (EPL) 1st/2nd compartment intersection 2nd/3rd compartment intersection (ECRL and ECRB/EPL) Radial artery/1st compartment Radial artery/2nd/3rd compartment intersection (ECRL and ECRB/EPL)
**Extension**	1st compartment (APL and EPB) 3rd compartment (EPL) 1st/2nd compartment intersection 2nd/3rd compartment intersection (ECRL and ECRB/EPL) Radial artery/1st compartment Radial artery/2nd/3rd compartment intersection (ECRL and ECRB/EPL)	Thenar muscles (as above) 1st dorsal interosseous/FPL/FPB/AdP Median nerve/FPL Median nerve/FPL/FCR/radial artery Median nerve (TCL, paraneural sheath, and interfascicular epineurium) Median nerve/TCL Palmar radiocarpal and palmar intercarpal ligaments/median nerve
**Abduction**	1st compartment (APL and EPB) 1st/2nd compartment intersection (APL, EPB/ECRL, and ECRB) Radial artery/1st compartment	Adductor pollicis Thenar muscles (as above) 1st dorsal interosseous/FPL/FPB/AdP
**Common** (all directions)	Extensor retinaculum Deep palmar arch Metacarpal/trapezium (CMC joint)	

**Table 4:** The 15-point FHR treatment protocol for five thumb pain conditions.

Point	Target Structure	Compartment/Region	Key Indication
1	APL/EPB sheath and retinaculum	1st extensor compartment	de Quervain’s disease
2	EPL/Lister’s tubercle	3rd extensor compartment	Thumb extension pain
3	APL-EPB/ECRL-ECRB intersection	1st–2nd compartment crossing	Intersection syndrome
4	EPL/ECRB-ECRL intersection	2nd–3rd compartment crossing	Wrist–thumb combined pain
5	Radial artery/1st compartment crossing	Radial styloid (vascular crossing)	Vascular–fascial entrapment
6	Radial artery/EPL crossing	Anatomical snuffbox (vascular crossing)	Radial artery–EPL entrapment
7	1st dorsal interosseous/adductor pollicis/deep palmar arch; FPL	First web space (Hegu)	Deep thumb pain
8	Adductor pollicis (oblique + transverse heads)	Deep thenar	Adduction pain; grip weakness
9	Dorsal and palmar interossei/deep palmar arch	Deep palm	Deep hand pain
10	APB/opponens pollicis/FPB/metacarpal	Thenar eminence	Thumb mobility restriction
11	TCL/median nerve	Carpal tunnel	Carpal tunnel syndrome
12	Paraneural sheath/interfascicular epineurium	Carpal tunnel (intraneural)	CTS (internal entrapment)
13	Median nerve/FPL/FCR interface	Distal palmar wrist (proximal to TCL)	CTS; forearm pain
14	Median nerve/FPL (carpal tunnel)	Carpal tunnel (deep)	CTS; thumb pain
15	Palmar radiocarpal and palmar intercarpal ligaments/median nerve	Deep carpal tunnel (deep to TCL)	CTS (adjunct)

**Table 5:** Supplementary Videos list and 15-point protocol correspondence.

Video	Protocol Point	Content Description
S1	Point 1	First Extensor Compartment Release (de Quervain)
S2	Point 2	Third Extensor Compartment Release (EPL)
S3	Point 3	First–Second Compartment Intersection Release
S4	Point 4	Second–Third Compartment Intersection Release
S5	Point 5	Radial Artery/First Compartment Crossing Release
S6	Point 6	Radial Artery/EPL Crossing Release
S7	Point 7	Gokoku (Hegu) Release (First Dorsal Interosseous/Adductor Pollicis/Deep Palmar Arch; FPL)
S8	Point 8	Adductor Pollicis Release
S9	Point 9	Deep Palmar Arch/Interossei Release
S10	Point 10	Thenar Muscles (APB/Opponens/FPB) Release
S11	Point 11	Median Nerve/Transverse Carpal Ligament Release
S12	Point 12	Paraneural Sheath/Interfascicular Epineurium Release
S13	Point 13	Median Nerve/FPL/FCR Interface
S14	Point 14	Median Nerve/Flexor Pollicis Longus Release
S15	Point 15	Palmar Radiocarpal and Palmar Intercarpal Ligaments/Median Nerve Release

**Table 6:** Evidentiary basis for each POINT (see Section 3.1).

Point	Anatomical Mapping	Sonographic Observation	Clinical Consensus	Published Literature
1	✓	✓	✓	✓ [17,21]
2	✓	✓	✓	—
3	✓	✓	✓	—
4	✓	✓	✓	—
5	✓	✓	✓	—
6	✓	✓	✓	—
7	✓	✓	✓	✓ [25]
8	✓	✓	✓	—
9	✓	✓	✓	—
10	✓	✓	✓	—
11	✓	✓	✓	✓ [23,27,30]
12	✓	✓	✓	✓ [27]
13	✓	✓	✓	—
14	✓	✓	✓	—
15	✓	✓	✓	✓ [28,29]

✓, evidence available in this category; —, no evidence available in this category. Numbers in brackets indicate the corresponding references.

**Table 7:** Comparison of thumb pain management modalities (see Section 4.2).

Modality	Primary Indication	Proposed Mechanism	Evidence Level	Position in Current Protocol
Rehabilitation/physical and exercise therapy	Most thumb pain conditions (first-line)	Movement patterns, strength, and functional load	High (RCTs for individual conditions)	Not replaced by US-FHR; US-FHR may be adjunct/preparatory
Corticosteroid injection	de Quervain’s, CMC OA, and CTS	Inflammatory modulation	High (short-to-intermediate)	Distinct mechanistic axis from US-FHR; potentially complementary
Surgical release (first dorsal compartment release, arthroplasty, CTR, and debridement)	Refractory/structural pathology	Mechanical release of established anatomical constraint	High for refractory cases	US-FHR proposed as intermediate step before surgical escalation
Interfascial injection (MPS RCT support) [48]	Myofascial pain syndrome (component)	Fascial layer separation and HA-related densification reduction	Prospective, randomized, and double-blinded RCT [48]	Supports MPS component of the 15-point protocol
15-point US-FHR (proposed)	Five converging thumb pain conditions with fascial densification	Mechanical separation, HA dilution, and mechanotransduction reset (hypothesized)	Anatomical rationale + clinical experience; controlled trials pending	Central to the proposed clinical protocol; validation required

## Data Availability

No new datasets were generated or analyzed for this clinical protocol article. The 15 supplementary procedural videos (Videos S1–S15) demonstrating the ultrasound-guided fascia hydrorelease techniques are publicly available at Zenodo (Repository: Zenodo; DOI: 10.5281/zenodo.20485196; URL: https://doi.org/10.5281/zenodo.20485196), under a Creative Commons Attribution 4.0 International (CC BY 4.0) license.

## Supplementary Materials

The following supporting information is deposited at Zenodo (DOI: 10.5281/zenodo.20485196; URL: https://doi.org/10.5281/zenodo.20485196): The FHR procedure for each of the 15 protocol points is demonstrated in the following 15 supplementary videos: Video S1: Point 1—first extensor compartment release (de Quervain); Video S2: Point 2—third extensor compartment release (EPL); Video S3: Point 3—intersection of the first and second compartments; Video S4: Point 4—intersection of the second and third compartments; Video S5: Point 5—radial artery/first compartment crossing; Video S6: Point 6—radial artery/EPL-ECRB-ECRL crossing; Video S7: Point 7—first dorsal interosseous/adductor pollicis/deep palmar arch; Video S8: Point 8—adductor pollicis; Video S9: Point 9—deep palmar arch/dorsal and palmar interossei; Video S10: Point 10—thenar muscles (APB/opponens pollicis/FPB); Video S11: Point 11—median nerve/transverse carpal ligament; Video S12: Point 12—paraneural sheath/interfascicular epineurium; Video S13: Point 13—median nerve/FPL/FCR interface; Video S14: Point 14—median nerve/flexor pollicis longus (carpal tunnel); Video S15: Point 15—palmar radiocarpal and palmar intercarpal ligaments/median nerve.

## Abbreviations

APB, abductor pollicis brevis; AdP, adductor pollicis; APL, abductor pollicis longus; CMC, carpometacarpal; CPPD, calcium pyrophosphate deposition disease; CTS, carpal tunnel syndrome; ECM, extracellular matrix; ECRB, extensor carpi radialis brevis; ECRL, extensor carpi radialis longus; EPB, extensor pollicis brevis; EPL, extensor pollicis longus; FCR, flexor carpi radialis; FHR, fascia hydrorelease; FPB, flexor pollicis brevis; FPL, flexor pollicis longus; HA, hyaluronic acid; IP, interphalangeal; JNOS, Japanese Non-Surgical Orthopedics Society; MCP, metacarpophalangeal; MMP, matrix metalloproteinase; MPS, myofascial pain syndrome; NSAID, non-steroidal anti-inflammatory drug; OA, osteoarthritis; OP, opponens pollicis; TAZ, transcriptional co-activator with PDZ-binding motif; TCL, transverse carpal ligament; TGF-β, transforming growth factor-beta; TIMP, tissue inhibitor of metalloproteinases; TPS, Thumb Pain Syndrome (proposed as a hypothesis; see Section 4.3, Limitations); UCL, ulnar collateral ligament; US-FHR, ultrasound-guided fascia hydrorelease; WHAT, wrist hyperflexion and abduction of the thumb; YAP, Yes-associated protein.
